# Systematic review of the effect of caffeine therapy effect on cardiometabolic markers in rat models of the metabolic syndrome

**DOI:** 10.1186/s12902-023-01288-4

**Published:** 2023-02-06

**Authors:** Isa Abdulla Alhadi, Ahmed Mohammed Al Ansari, Aseel Fuad Fahad AlSaleh, Ahmed M. Abdulla Alabbasi

**Affiliations:** Department of Gifted Education, Arabian Gulf University, P.O. Box: 26671, Manama, Bahrain

**Keywords:** HDL-C, Hypertension, Obesity, Insulin resistance, Dyslipidemia

## Abstract

This systematic review aimed to study caffeine's effect on the cardiometabolic markers of the metabolic syndrome and to evaluate caffeine’s application as a potential therapeutic agent in rat models. The systematic review was structured and synthesized according to Preferred Reporting Items for Systematic Reviews and Meta-Analyses (PRISMA) and the Population, Intervention, comparator, outcome (PICO) framework. A literature search was conducted in PubMed, Scopus, and ScienceDirect to identify studies that used caffeine as an intervention in the rat model of the metabolic syndrome or any of its components compared with no treatment or controls. Studies that did not mention the disease manifestations from the experimental model used, without rat subjects, and which induced renovascular hypertension were excluded. The risk of bias in the included studies was assessed using the Systematic Review Center for Laboratory Animal Experimentation risk-of-bias tool. The main outcomes assessed were caffeine’s effect on obesity, dyslipidemia, hepatic steatosis, hepatic dysfunction, insulin resistance, and hypertension. Out of 228 studies retrieved from the search, 18 met our inclusion criteria and were included in the systematic review. Caffeine was found to favorably reduce obesity and insulin resistance in the rat model of the metabolic syndrome. The effects of caffeine on dyslipidemia, hepatic steatosis, hepatic dysfunction, and hypertension remain inconclusive. The main limitations of this study are the heterogeneity of the included studies in terms of the disease model used, experimental duration, methods to assess outcomes, including studies that were only published in English, measurement units used, and graphical data without and numerical mention in the results section. As a result, quantitative synthesis was unfeasible, and a qualitative descriptive synthesis was conducted; this might have led to the under characterization of caffeine’s effect on metabolic syndrome and its potential as an adjuvant therapy in metabolic syndrome. Caffeine has favorable effects on the metabolic syndrome, chiefly reducing obesity and insulin resistance. Future research is encouraged to delve into caffeine’s effect on dyslipidemia, hepatic steatosis, hepatic dysfunction, and hypertension, which is necessary if caffeine is to be used as a potential clinical adjuvant therapy to treat the metabolic syndrome.

**Supplementary Information:**

The online version contains supplementary material available at 10.1186/s12902-023-01288-4.

## Introduction

The coexistence of multiple established cardiovascular risk factors, such as obesity, insulin resistance, dyslipidemia, and hypertension, is clinically recognized as the metabolic syndrome [1]. In the United States, the prevalence of the metabolic syndrome is estimated as 34.2% [2]. Therefore, the metabolic syndrome represents a considerable disease burden for a significant segment of the population, particularly as individuals with the metabolic syndrome tend to have more frequent hospitalizations, higher healthcare expenses, and higher rates of outpatient service utilization [3]. Consequently, a novel therapeutic agent, such as caffeine, is required to adequately treat the metabolic syndrome and to reduce the considerable health and financial costs that affected patients incur. Caffeine's effects on the metabolic syndrome and its components were previously documented from preclinical research and included favorable effects, such as a reduction in blood glucose and serum insulin concentrations that resulted in better glycemic control and a reduction in insulin resistance, in rat models of the metabolic syndrome [4]. Furthermore, caffeine therapy ameliorated hypertension, as indicated by a drop in the mean arterial blood pressure [4]. In experimental rat models, caffeine hindered the growth of visceral fat deposits and increase in bodyweight, which are associated with obesity [4]. However, to our knowledge, no systematic review has focused solely on caffeine's metabolic effects in a rat model of the metabolic syndrome. Therefore, this systematic review was conducted with the aim to study the effect of caffeine on the cardiometabolic markers of the metabolic syndrome and evaluate the feasibility of using caffeine as a potential therapeutic agent in the rat model of the metabolic syndrome.

## Methods

This systematic review was structured according to Preferred Reporting Items for Systematic Reviews and Meta-Analyses (PRISMA) [5] and the Population, Intervention, Comparator, and Outcome (PICO) framework [6]. This review was not registered prior to its writing.

### Eligibility

Studies were eligible for inclusion in this review if they matched all of the following criteria: 1) experimental study design; 2) included only rats with the metabolic syndrome or any of its components (atherosclerosis, dyslipidemia, diabetes mellitus, obesity, or hypertension) or nonalcoholic fatty liver disease as subjects; 3) caffeine was the only interventional compound in at least one of the experimental groups; 4) contained at least one of the following key outcomes: serum cholesterol level, serum insulin level, serum glucose level, blood pressure, serum triglyceride level, liver cholesterol level, liver triglyceride level, and results of oral glucose tolerance tests (OGTT); 5) articles in English that were published from database inception until September 14, 2020. Studies were excluded if they did not specify the disease manifestations in the experimental model.

### Search strategy

The search was run in the following databases: PubMed, Scopus, and ScienceDirect. The following terms and their equivalent medical subject headings terms were used: “caffeine”, “Metabolic Syndrome”, “Rats”, “Mice”, “Atherosclerosis”, “Dyslipidemia”, “Diabetes Miletus”, and “Non-Alcoholic Fatty Liver Disease”. The term “mice” was included to broaden the scope of the search and to identify studies that used mixed rodent species as subjects. The last search was conducted in the second week of September 2020. The search results were then exported to the reference manager Endnote X7 [7]. For a detailed view of the search strategy terms, refer to the Supplementary Data.

### Selection

Two independent reviewers manually screened the title, abstract, and full-text articles of studies for inclusion in accordance with the eligibility criteria. Disagreements, if any, were resolved through consensus. In addition, the references of the full-text studies that were included were screened for inclusion in the review.

### Data collection

Two reviewers independently extracted data manually from each study into a Microsoft Excel 2018 sheet, which was then cross-checked for accuracy, and disagreements were resolved through consensus. Data items included bibliographic data (author, publishing year, and journal), participant data (strain, sex, age, and initial bodyweight), study design (duration, disease model, disease symptoms that were present, name of the diet, dietary macronutrients, diet ingredients, diet availability, name of the experimental group, number of experimental groups, and subject allocation method), and intervention data (dose, administration method, administration duration, and age at administration). Outcome measures that were collected were classified as those pertaining to the effect of caffeine on the components of the metabolic syndrome, including obesity (food intake, energy intake, final body weight, change in body weight, body fat percentage, whole-body fat weight, whole-body white adipose tissue weight, and body fat-pad weight); dyslipidemia (levels of serum triglycerides, serum total cholesterol, serum low density lipoprotein cholesterol [LDL-C], serum high density lipoprotein cholesterol [HDL-C], and serum non-esterified fatty acids); hepatic steatosis (levels of liver triglycerides and liver cholesterol and liver weight); hepatic dysfunction (levels of serum aspartate transaminase [AST], serum alanine transaminase [ALT], serum alkaline phosphatase [AP], serum lactate dehydrogenase [LDH], serum albumin, and serum total bilirubin); insulin resistance (levels of serum fasting glucose, serum postprandial glucose, urinary glucose, serum fasting insulin, and serum postprandial insulin; area under the glucose curve; area under the insulin curve; fluid intake; urinary volume; OGTT; and insulin tolerance test [ITT]); and hypertension (systolic blood pressure [SBP], diastolic blood pressure [DBP], and mean arterial blood pressure [MAP]). During data collection, only the latest result was extracted for synthesis in the review. During the collection of data from the study by Suzuki et al., [8] we used MedCalc software to make an adjustment by calculating the significance level of serum non-esterified fatty acids, serum fasting glucose, and serum fasting insulin [9].

### Bias assessment

Risk-of-bias assessment was independently performed by 2 reviewers using the Systematic Review Center for Laboratory Animal Experimentation (SYRCLE) risk-of-bias tool [10]. The tool consists of several bias domains including selection bias (random sequence generation, baseline characteristics, and allocation concealment), performance bias (random housing and blinding of participants and personnel), detection bias (random outcome assessment and blinding of outcome assessment), attrition bias (incomplete outcome data), reporting bias (selective reporting), and other bias with signaling questions for each domain. Disagreements between reviewers were resolved through consensus. Finally, a risk-of-bias summary and graph were generated using RevMan 5.4 [11].

### Data analysis and synthesis

Due to the heterogeneity of data across studies, data analysis was limited to the description of qualitative data, which resulted in an inability to perform a meta-analysis. Studies were deemed eligible for outcome synthesis based on whether they reported the required outcome, either through means and standard deviations or through visual graphs. When synthesizing data from graphs, we analyzed outcomes based on whether it was higher or lower than the control group by using visual indicators on study graphs and the author’s in-text description of the results and their significance. However, the mean and standard deviation, if available, were preferred over graphical data in the synthesis process. Additionally, a table summarizing the outcomes, outcome measures, and results was created based on whether the caffeine group had significantly or insignificantly lower or higher values than the control group (*p*˂0.05 was set as the level of significance) for that specific measure. Furthermore, the number of studies and the reported outcome measures were included along with the reported results.

## Results

### Study selection

**Fig. 1:**
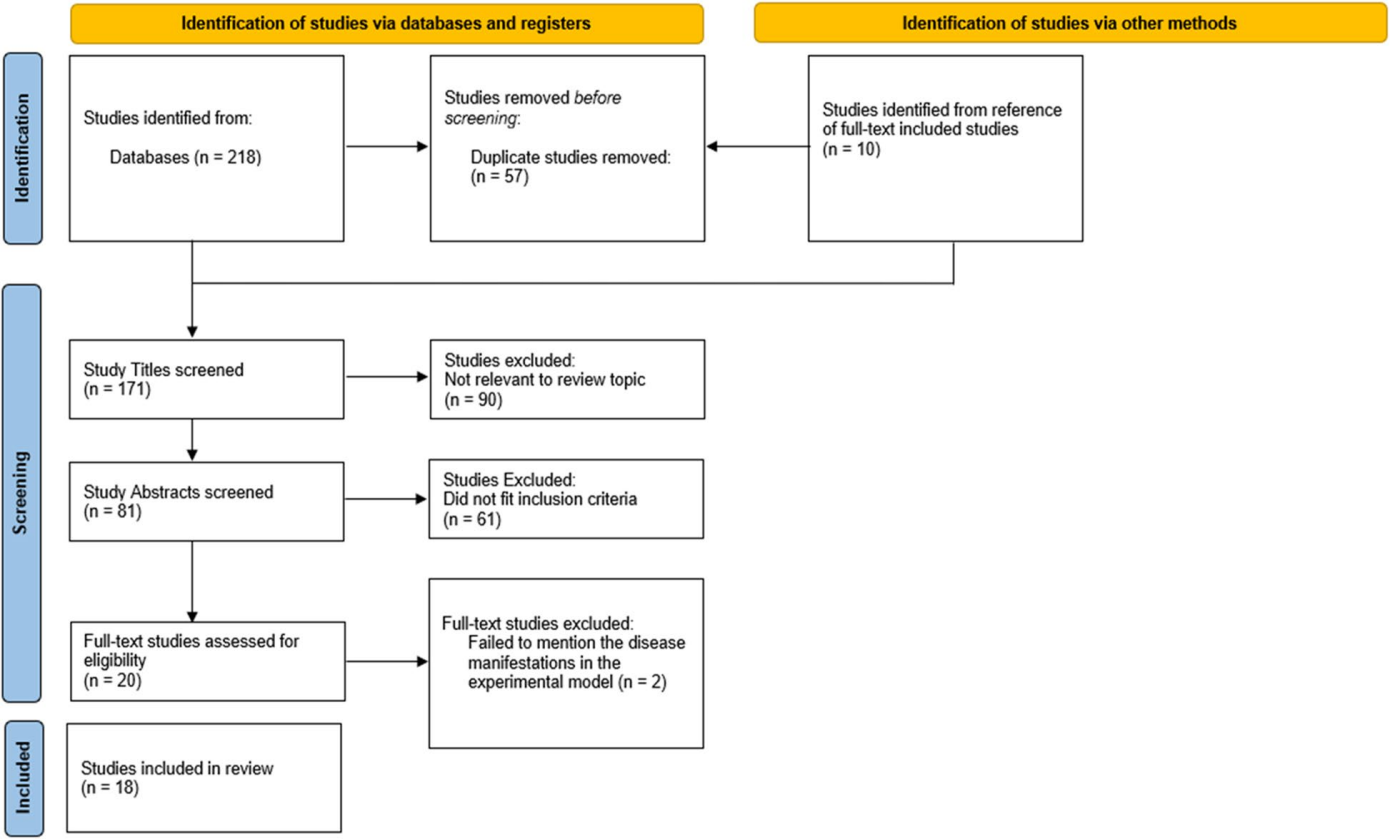
PRISMA flow diagram of study selection in this review

In total, 228 papers were retrieved, of which 218 were identified from the database searches and 10 were selected manually from the references of the studies that were included; 57 duplicate studies were removed automatically using the reference manager Endnote X7.8 [7], thereby decreasing the number of studies to 171. Of the 171 study titles that were screened, 90 were excluded for lack of relevance to the review topic. Among the abstracts of the remaining 81 studies that were screened, only 20 fulfilled the inclusion criteria and were included for full-text screening. From these 20 full-text studies, 2 studies [12, 13] were excluded as per the exclusion criteria for lack of information on disease manifestations that resulted from the experimental model. The PRISMA flow diagram of the study selection procedure is shown in Fig. 1. Finally, the remaining 18 studies [4, 8, 14–29] were included in the final qualitative synthesis (Table 1**)**, and a meta-analysis was unfeasible due to data heterogeneity and the poor accuracy of data extraction from charts.

**Table 1:** Characteristics of the studies that were included in this review

Author	Year	Study Title	Participant Rat Strain	Disease		Intervention			Control		Relevant Outcome Measures	Method of Administration
				Induction method	Phenotype	Dose	Duration	N	Placebo	N		
**Ohnishi A Fau—Branch, Branch Ra Fau—Jackson **^**14**^	1986	Chronic caffeine administration exacerbates renovascular, but not genetic, hypertension in rats	Spontaneously hypertensive rats Okamoto–Aoki strain	Genetic	Hypertension	0.1%	7 weeks	8	Drinking water without caffeine	8	Fluid intake, food intake, urinary volume, systolic blood pressure	In drinking water
**Sugiyama, Ohishi **^**15**^	1989	Comparison of the plasma cholesterol-elevating effects of caffeine and methionine in rats on a high-cholesterol diet	Wistar rats	High-cholesterol diet	Dyslipidemia	0.3%	2 weeks	7	Basal diet without caffeine	8	Food intake, change in body weight, serum total cholesterol, serum triglycerides, serum HDL-C, Liver triglycerides, liver cholesterol, liver weight	In feed
**Choi, Lee **^**16**^	1993	Chronic caffeine ingestion exacerbates 2-kidney, 1-clip hypertension and ameliorates deoxycorticosterone acetate-salt hypertension in rats	Sprague–Dawley rats	Deoxycorticosterone acetate 200 mg/kg, subcutaneous implant, + salt hypertension (0.9% NaCl intake)	Hypertension	0.1%	24 days	6	Drinking solution (0.9% NaCl) without caffeine	6	Systolic blood pressure	Drinking solution with 0.9% NaCl
**Tofovic and Jackson **^**17**^	1999	Effects of long-term caffeine consumption on renal function in spontaneously hypertensive heart failure prone rats	lean spontaneously hypertensive heart failure (SHHF/Mcc-fa^cp^) rats	Genetic	Hypertension and heart failure	0.1%	20 weeks	10	Drinking tap water without caffeine	9	Systolic blood pressure, diastolic blood pressure, mean arterial pressure	In drinking water
**Tanner and Tanner **^**18**^	2001	Chronic caffeine consumption exacerbates hypertension in rats with polycystic kidney disease	Heterozygous Han: Sprague–Dawley (Han: SPRD) rat	Genetic	Hypertension and autosomal dominant polycystic kidney disease	0.1 mg/mL	20 weeks	6	Drinking tap water without caffeine	17	Fluid intake, final body weight, mean arterial blood pressure	In drinking water
						0.2 mg/mL		5				
**Tofovic, Kusaka **^**19**^	2001	Renal and metabolic effects of caffeine in obese (fa/facp), diabetic, hypertensive ZSF1 rats	Obese (fa/fa^cp^) ZSF1 rat	Genetic	Obesity Hypertension Type 2 diabetes Hypercholesterolemia Hypertriglyceridemia Renal dysfunction	0.1%	8 weeks	7	Drinking tap water without caffeine	8	Fluid intake, food intake, urinary volume, final body weight, serum triglycerides, serum total cholesterol, serum fasting glucose, serum postprandial glucose, area under the glucose curve, serum fasting insulin, serum postprandial insulin, area under the insulin curve, urinary glucose, systolic blood pressure, diastolic blood pressure, mean arterial blood pressure	In drinking water
**Tofovic, Kost **^**20**^	2002	Long-term caffeine consumption exacerbates renal failure in obese, diabetic, ZSF1 (fa-facp) rats	Obese (fa/fa^cp^) ZSF1 rat	Genetic	Obesity Hypertension Type 2 diabetes Hyperlipidemia Renal dysfunction	0.1%	30 weeks	12	Drinking tap water without caffeine	12	Fluid intake, food intake, urinary volume, final body weight, serum triglycerides, serum total cholesterol, serum fasting glucose, serum fasting insulin, urinary glucose, OGTT, systolic blood pressure, diastolic blood pressure, mean arterial blood pressure	In drinking water
**Park, Jang **^**21**^	2007	Long-term consumption of caffeine improves glucose homeostasis by enhancing insulinotropic action through islet insulin/insulin-like growth factor 1 signaling in diabetic rats	Sprague–Dawley rats	High-fat diet + 90% pancreatectomy	Type 2 diabetes	0.01%	12 weeks	9	Drinking water without caffeine	10	Fluid intake, energy intake, final body weight, body fat-pad weight, serum fasting glucose, serum fasting insulin	In drinking water
**Tofovic, Salah **^**22**^	2007	Early renal injury induced by caffeine consumption in obese, diabetic ZSF1 rats	Obese (fa/fa^cp^) ZSF1 rat	Genetic	Obesity Hypertension Hyperlipidemia Diabetes Renal dysfunction	0.1%	9 weeks	9	Drinking tap water without caffeine	10	Fluid intake, food intake, urinary volume, final body weight, serum triglycerides, serum total cholesterol, area under the glucose curve, serum fasting insulin, urinary glucose, OGTT, mean arterial blood pressure	In drinking water
**Kagami, Morita **^**23**^	2008	Protective effect of caffeine on streptozotocin-induced beta-cell damage in rats	Wistar rats	Streptozocin 65 mg/kg, intraperitoneal injection	Diabetes	10 mg/kg	1 week	-	Streptozocin and saline injection	-	Final body weight, serum fasting glucose, serum fasting insulin, OGTT	Intraperitoneal injection
						50 mg/kg						
						100 mg/kg						
**Conde, Nunes da Silva **^**4**^	2012	Chronic caffeine intake decreases circulating catecholamines and prevents diet-induced insulin resistance and hypertension in rats	Wistar rats	High-fat diet	Metabolic syndrome	1 g/L	15 days	12–15	Drinking water without caffeine	9 to 12	Fluid intake, food intake, change in body weight, body fat-pad weight, serum non-esterified fatty acids, serum fasting glucose, serum fasting insulin, mean arterial blood pressure	In drinking water
				High-sucrose diet	Insulin resistance Hypertension	1 g/L	15 days	12–15	Drinking water without caffeine	9 to 12	Fluid intake, food intake, change in body weight, body fat-pad weight, serum non-esterified fatty acids, serum fasting glucose, serum fasting insulin, mean arterial blood pressure	In drinking water
**Panchal, Wong **^**24**^	2012	Caffeine attenuates metabolic syndrome in diet-induced obese rats	Wistar rats	High-carbohydrate, high-fat diet	Metabolic syndrome Metabolic abnormalities Cardiovascular remodeling Nonalcoholic steatohepatitis	0.5 g/kg of food or 47.9 ± 1.0 mg/kg/day	8 weeks	10	Fed experimental diet without caffeine	10	Fluid intake, food intake, energy intake, final body weight, whole-body fat weight, body fat-pad weight, serum triglycerides, serum total cholesterol, serum non-esterified fatty acids, serum AST, serum ALT, serum AP, serum LDH, serum albumin, serum total bilirubin, liver weight, serum fasting glucose, area under the glucose curve, serum fasting insulin, OGTT, ITT, systolic blood pressure	In feed
**Naidoo and Islam **^**25**^	2014	Development of an alternative non-obese non-genetic rat model of type 2 diabetes using caffeine and streptozotocin	Sprague–Dawley rats	Streptozocin 65 mg/kg, intraperitoneal injection	Diabetes	20 mg/kg	13 weeks	10	Injection containing only normal saline (0.9% NaCl)	10	Fluid intake, food intake, final bodyweight, serum triglycerides, serum total cholesterol, LDL-C, HDL-C, serum AST, serum ALT, serum AP, serum LDH, liver weight, serum fasting glucose, serum postprandial glucose, serum fasting insulin, OGTT	Single injection
						40 mg/kg		10				
**Xu, Zhang **^**26**^	2015	The anti-obesity effect of green tea polysaccharides, polyphenols and caffeine in rats fed with a high-fat diet	Sprague–Dawley rats	High-fat diet	Obesity	400 g/kg	6 weeks	12	Fed experimental diet plus saline	12	Final body weight, body fat percentage, whole-body fat weight, serum triglyceride, serum total cholesterol, LDL-C, HDL-C	In feed
**Kumbhar, Une **^**27**^	2016	Exaggeration of type 2 diabetes due to caffeine-nicotine coadministration: A study in rats	Sprague–Dawley rats	High-fat diet for 2 weeks prior to a single intravenous streptozocin injection (50 mg/kg)	Diabetes	40 mg/kg	3 weeks	10	Injected with sodium chloride in distilled water	10	Change in body weight, serum total cholesterol, LDL-C, HDL-C, serum AST, serum ALT, serum fasting glucose	Intraperitoneal injection
**Suzuki, Shindo **^**8**^	2017	Combined long-term caffeine intake and exercise inhibits the development of diabetic nephropathy in OLETF rats	Otsuka Long–Evans Tokushima fatty (OLETF) rats	Genetic	Obesity Diabetes	0.2% (90.7 ± 4.7 mg/kg/day)	4 weeks	8	Fed experimental diet without caffeine	8	Food intake, urinary volume, final body weight, serum non-esterified fatty acids, serum fasting glucose, serum fasting insulin, systolic blood pressure, diastolic blood pressure	In feed
**Helal, Ayoub **^**28**^	2018	Caffeine affects HFD-induced hepatic steatosis by multifactorial intervention	Wistar rats	High-fat diet	Nonalcoholic fatty liver disease	20 mg/kg/day	16 weeks	10	Received vehicle only	10	Serum triglycerides, serum total cholesterol, HDL-C, serum AST, serum ALT, serum albumin, serum total bilirubin	Orally
						30 mg/kg/day		10				
**Yang, Zhu **^**29**^	2019	Coadministration of epigallocatechin-3-gallate (EGCG) and caffeine in low dose ameliorates obesity and nonalcoholic fatty liver disease in obese rats	Sprague–Dawley rats	High-fat diet	Obesity Nonalcoholic fatty liver disease	20 mg/kg	4 weeks	8	Oral gavage with distilled water	8	Food intake, energy intake, final body weight, body fat percentage, white adipose tissue weight, body fat-pad weight, serum triglycerides, serum total cholesterol, LDL-C, HDL-C, serum non-esterified fatty acids, serum AST, serum ALT	Oral gavage

*AST* aspartate transaminase, *ALT* alanine transaminase, *AP* alkaline phosphatase, *HDL-C* high density lipoprotein cholesterol; insulin tolerance test, *ITT LDH* lactate dehydrogenase, *LDL-C* low density lipoprotein cholesterol, *OGTT* oral glucose tolerance test

### Risk-of-bias assessment

**Fig. 2:**
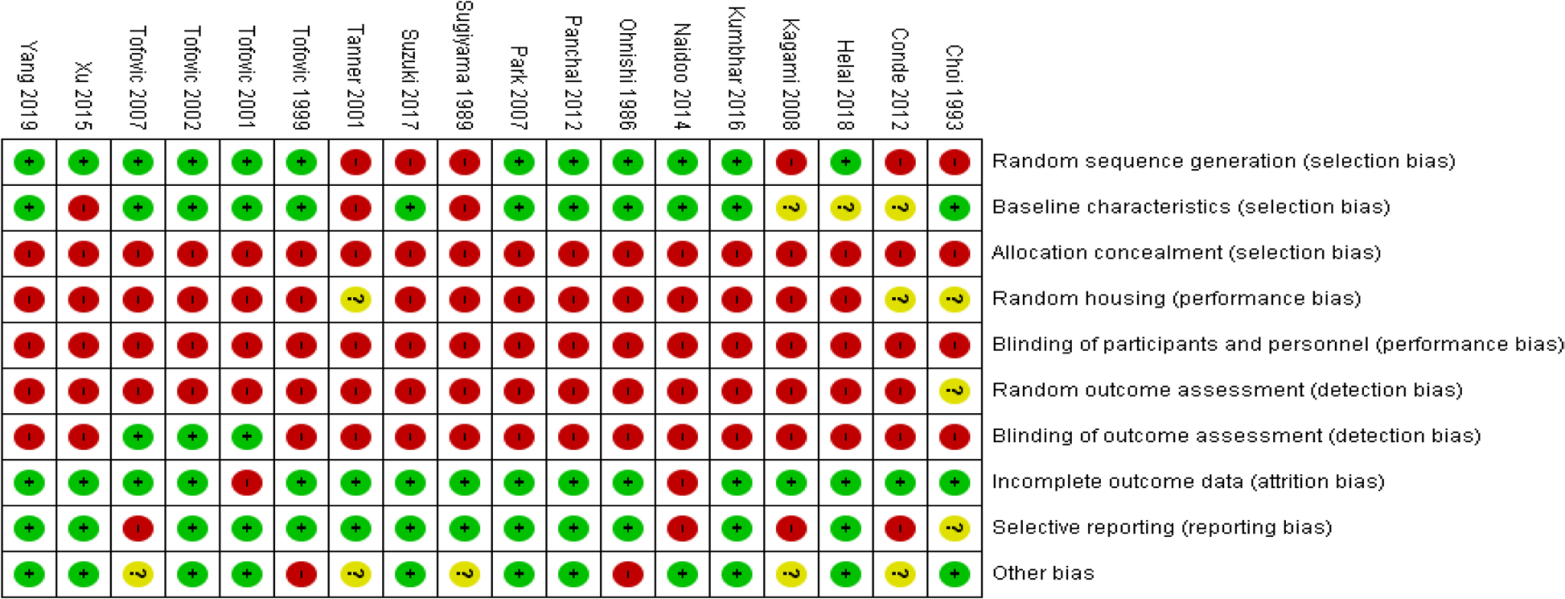
Risk of bias summary. Green = low risk of bias; Red = high risk of bias; Yellow = unclear risk of bias

In the random sequence generation assessment, 12 studies [14, 17, 19–22, 24–29] had a low risk of bias without specifying the method, whereas the remaining 6 studies [4, 8, 15, 16, 18, 23] had a high risk of bias due to the nonrandomized group allocation. In the assessment of baseline characteristics, 12 studies [14, 16, 17, 19–22, 24, 25, 27, 29] had a low risk of bias, 3 had a high risk of bias [15, 18, 26], and 3 had an unclear risk of bias [4, 23, 28]. In the assessment of allocation concealment, all of the studies that were included [4, 8, 14–29] had a high risk of bias due to lack of blinding of the investigator during group allocation. In the assessment of random housing, 15 studies [8, 14, 15, 17, 19–29] had a high risk of bias and 3 studies [4, 16, 18] had an unclear risk of bias. With respect to the blinding of participants and personnel, all 18 studies had a high risk of bias due to the lack of blinding of investigators. In the assessment of random outcome, 17 studies [4, 8, 14, 15, 17–29] had a high risk of bias and 1 study [16] had an indeterminate risk of bias. In the assessment of blinding of outcome, 3 studies [19, 20, 22] had a low risk of bias and 15 studies [4, 8, 14–18, 21, 23–29] had a high risk of bias. In the incomplete outcome data assessment, 16 studies [4, 8, 14–18, 20–24, 26–29] had a low risk of bias and 2 studies [19, 25] had a high risk of bias. In the assessment of selective reporting, 13 studies [8, 14, 15, 17–21, 24, 26–29] had a low risk of bias, 4 studies [4, 22, 23, 25] had a high risk of bias, and one study [16] had an indeterminate risk of bias. In other bias assessments, 11 studies [8, 16, 19–21, 24–29] had a low risk of bias, 2 studies [14, 17] had a high risk of bias, and 5 studies [4, 15, 18, 22, 23] had an indeterminate risk of bias. The risk-of-bias summary is shown in Fig. 2.

### Characteristics of the included studies

Among the 18 studies that were included, 6 used Sprague–Dawley rats [16, 21, 25–27, 29], 5 used Wistar rats [4, 15, 23, 24, 28], 3 used Obese (fa/fa^cp^) ZSF1 rats [19, 20, 22], and one study each used Spontaneously Hypertensive rat Okamoto–Aoki strain [14], Lean Spontaneously Hypertensive Heart Failure (SHHF/Mcc-fa^cp^) rats [17], Heterozygous Han: Sprague–Dawley (Han:SPRD) rats [18], and Otsuka Long–Evans Tokushima fatty (OLETF) rats [8]. For disease induction, 7 studies used genetic methods [8, 14, 17–20, 22], 4 used high-fat diets [4, 26, 28, 29], 2 used streptozocin (65 mg/kg via intraperitoneal injection) [23, 25], and 1 study each used a high-cholesterol diet [15], deoxycorticosterone acetate (200 mg subcutaneous implant) + salt hypertension (0.9% NaCl drinking) [16], high-fat diet + 90% pancreatectomy [21], high-sucrose diet [4], high-carbohydrate high-fat diet [24], and a high-fat diet for 2 weeks prior to streptozocin (50 mg/kg) intravenous injection [27]. Notably, the study by Conde et al. [4] simultaneously used 2 independent disease models, with each comprising an interventional and control group, and is therefore referred to by its 2 independent experimental models – high-fat diet model (HFDM) [4] or high-sucrose diet model (HSDM) [4] – throughout the review. For a detailed description of the disease phenotype, interventional dose, duration, and method of administration in all the studies that were included, see Table 1. For more details on diet, age, and sex of rats used in the studies, refer to the Supplementary Data.

#### Effect of caffeine vs. control on obesity

With respect to obesity, 10 studies measured food intake, and 5 studies [15, 19, 20, 22, 29] reported a significantly lower food intake in the caffeine group than in the control group; among these, the most significant results were reported in the studies by Tofovic et al. (49 ± 3 g/kg of BW/d vs. 61 ± 1 g/kg of BW/d, *p*˂0.001) [20] and Wang et al. (25.8 ± 2 g/d vs. 28.1 ± 2.1 g/d, *p*˂0.01) [29]. Two studies [8, 24] and the study that used HSDM [4] reported an insignificantly higher food intake in the caffeine group than in the control group, whereas 2 studies [14, 25] and the study that used HFDM [4] reported an insignificantly lower food intake in the caffeine group than in the control group. Moreover, 3 studies measured energy intake, and 1 study [29] reported a significantly lower energy intake in the caffeine group than in the control group (111.7 ± 8.9 kcal/d vs. 121.5 ± 8.9 kcal/d, *p*˂0.01, respectively [29]); however, 1 study [24] reported an insignificantly higher energy intake in the caffeine group than in the control group; and 1 study [21] reported an insignificantly lower energy intake in the caffeine group than in the control group. Furthermore, in 11 studies, the final body weight was measured, and 1 study [18] reported a significantly higher final body weight in the caffeine (0.1 and 0.2 mg/mL, respectively) group than in the control group (499 ± 26 g and 508 ± 14 g vs. 462 ± 29 and 462 ± 29 g, *p*˂0.05 and < 0.01, respectively) [18]; however, 8 studies [8, 19–22, 24, 26, 29] reported a significantly lower final body weight in the caffeine group than in the control group, of which the most significant were the study by Tofovic et al. (650 ± 20 g vs. 740 ± 11 g, *p*˂0.001) [20] and Suzuki, Shindo [8] (*p*˂0.001). One study each reported an insignificantly higher [25] and insignificantly [23] lower final body weight in the caffeine group than in the control group, respectively. Among the 3 studies that measured the change in body weight, 1 study [27] reported a significantly higher change in body weight in the caffeine group than in the control group (− 83 ± 2.32 g vs. − 61 ± 3.31 g, *p* < 0.05, respectively) [27]. One study [15] and another study that used HFDM [4] reported a significantly lower change in bodyweight in the caffeine group than in the control group (60 ± 4 g/14 days vs. 80 ± 3 g/14 days [15] and 2.39 ± 0.36 g/day vs. 4.32 ± 0.45 g/day [4], *p* < 0.05 and *p* < 0.001, respectively). However, the study arm with HSDM [4] induced an insignificantly lower change in body weight in the caffeine group than in the control group. Among the 18 studies included in this review, 2 measured the body fat percentage, and both [26, 29] reported a significantly lower body fat percentage in the caffeine group than in the control group (1.81 ± 0.60% vs. 2.85 ± 0.45%, *p* < 0.05 [26] and 3.07 ± 0.65% vs. 4.99 ± 0.47%, *p* < 0.01) [29]. Similarly, 2 studies measured whole-body fat weight, and both [24, 26] reported a significantly lower whole-body fat weight in the caffeine group than in the control group (80 ± 6 g vs. 152 ± 7 g [24] and 5.22 ± 1.81 g vs. 10.99 ± 2.24 g [26], *p* < 0.05 for both). One study [29] measured white adipose tissue weight and reported a significantly lower white adipose tissue weight in the caffeine group than in the control group (17.3 ± 3.4 g vs. 29.3 ± 3.3 g, *p* < 0.01) [29]. Four studies measured body fat-pad weight but had considerable heterogeneity on which body fat-pad was harvested. Moreover, 3 of the 4 studies [21, 24, 29] that measured body fat-pad weight harvested epididymal fat and reported significantly lower body fat-pad weight in the caffeine group than in the control group (4.1 ± 0.5 g vs. 4.9 ± 0.6 g [21] and 122 ± 7 mg/mm tibial length vs. 225 ± 13 mg/mm tibial length [24], and 8.9 ± 1.5 g vs. 13.1 ± 2.5 g [29]; *p* < 0.05, *p* < 0.05, and *p* < 0.01, respectively). Only 1 [24] out of the 4 studies that measured body fat-pad weight harvested retroperitoneal, omental, and total abdominal (retroperitoneal + epididymal + omental) fat pads and found significantly lower body fat-pad weight in the caffeine group than in the control group (tibial length, 198 ± 10 mg/mm vs. 357 ± 21 mg/mm, 83 ± 6 mg/mm vs. 194 ± 12 mg/mm, and 402 ± 21 mg/mm vs. 775 ± 46 mg/mm, respectively; *p* < 0.05 for all) [24]. One each of the 4 studies that measured the body fat-pad weight harvested the perirenal fat [29] and visceral fat [4] reported significantly lower body fat-pad weight in the caffeine group (8.4 ± 2.3 g vs. 16.2 ± 3.6 g, *p* < 0.01) and the HFDM [4] caffeine group (7.80 ± 0.90 g/kg vs. 12.70 ± 0.64 g/kg, *p* < 0.001) [4] than in the control group, respectively; however, the body fat-pad weight was insignificantly lower in the HSDM [4] caffeine group than in the control group.

#### Effect of caffeine vs. control on dyslipidemia

Among the 9 studies that measured serum triglycerides, 3 [26, 28, 29] found significantly lower serum triglycerides in the caffeine group than in the control group (0.86 ± 0.16 mmol/L vs. 1.53 ± 0.22 mmol/L [*p* < 0.05] [26], 89.6 ± 9 and 50.6 ± 2.9 mg/dL [caffeine 20 and 30 mg/kg/day groups, respectively] vs. 131.8 ± 3 mg/dL [*p* < 0.05 for both] [28], and 1.05 ± 0.13 mmol/L vs. 1.39 ± 0.17 mmol/L [*p* < 0.01], respectively) [29]. Although 1 study [24] reported a significantly higher serum triglyceride level in the caffeine group than in the control group (1.5 ± 0.2 mmol/L vs. 1.0 ± 0.1 mmol/L, *p* < 0.05) [24], 5 studies [15, 19, 20, 22, 25] reported an insignificantly lower serum triglyceride level in the caffeine group than in the control group. Furthermore, 10 studies measured serum total cholesterol, and 5 [15, 19, 20, 22, 24] found that the serum total cholesterol level was significantly higher in the caffeine group than in the control group; among these, the most significant differences were observed in the studies of Tofovic, Kusaka [19] (2001; *p* < 0.001) and Tofovic et al. (2002; 628 ± 102 mg/dL vs. 225 ± 8 mg/dL in the caffeine and control groups, respectively, *p* < 0.005) [20]. Two studies [26, 28] reported a significantly lower serum total cholesterol in the caffeine group than in the control group (2.78 ± 0.36 mmol/L vs. 4.32 ± 0.88 mmol/L [26] and 83.6 ± 3 mg/dL [caffeine 20 mg/kg/day] and 81.2 ± 5.8 mg/dL [caffeine 30 mg/kg/day] vs. 110 ± 4.4 mg/dL, *p* < 0.05 for all) [28]. Three studies [25, 27, 29] reported an insignificantly lower serum total cholesterol level in the caffeine group than in the control group. Four studies measured LDL-C, of which 3 [25, 26, 29] reported a significantly lower LDL-C level (LDL-C in the figure in Naidoo and Islam [25], 0.94 ± 0.18 mmol/L vs. 3.04 ± 0.93 mmol/L [26], and 0.51 ± 0.05 mmol/L vs. 0.63 ± 0.07 mmol/L [29]; *p* < 0.05 for all) and 1 study [27] reported an insignificantly higher LDL-C level in the caffeine group than in the control group, respectively. Of the studies that quantified HDL-C, 2 [15, 28] reported a significantly higher HDL-C level (28 ± 2 mg/100 mL vs. 23 ± 1 mg/100 mL [15] and 52 ± 5.1 mg/dL [caffeine 20 mg/kg/day] [28] and 50.1 ± 5.1 mg/dL [caffeine 30 mg/kg/day] vs. 30.4 ± 1.9 mg/dL [28], *p* < 0.05 for both) and 3 studies [25–27] reported an insignificantly lower HDL-C level in the caffeine group than in the control group; however, 1 study [29] reported an insignificantly higher HDL-C level in the caffeine group than in the control group. Four studies measured serum non-esterified fatty acids, of which 2 studies [8, 29] and the HSDM study [4] reported a significantly lower serum non-esterified fatty acid level in the caffeine group than in the control group (0.87 ± 0.04 mEq/L vs. 1.88 ± 0.14 mEq/L, *p* < 0.001 [8]; 0.31 ± 0.05 mmol/L vs. 0.39 ± 0.05 mmol/L, *p* < 0.05 [29]; and 610.24 ± 41.06 µM vs. 940.62 ± 89.66 µM, *p* < 0.001, respectively) [4]; however, 1 study [24] reported a significantly higher serum non-esterified fatty acid level in the caffeine group than in the control group (5.1 ± 0.4 mmol/L vs. 2.8 ± 0.3 mmol/L, *p* < 0.05) [24]. The HFDM [4] arm revealed an insignificantly lower serum non-esterified fatty acid level in the caffeine group than in the control group.

#### Effect of caffeine vs. control on hepatic steatosis

One study [15] measured liver triglyceride and liver cholesterol levels and reported a significantly lower liver triglyceride level (40.7 ± 1.4 mg/g vs. 53.8 ± 2.9 mg/g, *p* < 0.05) [15] and an insignificantly higher liver cholesterol in the caffeine group than in the control group. Three studies measured the liver weight, of which 2 studies [15, 24] and the caffeine 20 mg/kg group of Naidoo and Islam [25] reported an insignificantly lower liver weight in the caffeine group than in the control group; the caffeine 40 mg/kg group of Naidoo and Islam [25] reported an insignificantly lower liver weight in the caffeine group than in the control group.

#### Effect of caffeine vs. control on hepatic dysfunction

Five studies evaluated serum AST, of which 3 studies [25, 27, 29] reported an insignificantly lower serum AST level and 2 studies [24, 28] reported a significantly lower serum AST level (80 ± 5 U/L vs. 102 ± 5 U/L, *p* < 0.05 [24] and 162 ± 11.7 U/L [caffeine 20 mg/kg/day group] [28] and 157.2 ± 5.7 U/L [caffeine 30 mg/kg/day group] vs. 224 ± 20.5 U/L, *p* < 0.05) [28] in the caffeine group than in the control group. Five studies measured serum ALT, and 3 of them [24, 28, 29] reported a significantly lower serum ALT level in the caffeine group than in the control group (42 ± 3 U/L vs. 55 ± 3 U/L [24], 67 + 2.1 U/L [caffeine 20 mg/kg/day group] [28] and 64 + 4 U/L [caffeine 30 mg/kg/day group] vs. 110.4 + 3.2 U/L [28], and 63.6 ± 6.1 U/L vs. 77.3 ± 11.5 U/L [29]; *p* < 0.05 for all). However, 1 study [27] reported an insignificantly higher serum ALT level and another study [25] reported an insignificantly lower serum ALT level in the caffeine group than in the control group. Two studies measured the serum AP, and 1 study [24] reported a significantly higher serum AP level in the caffeine group than in the control group (363 ± 20 U/L vs. 261 ± 18 U/L, *p* < 0.05) [24]. Naidoo and Islam [25] reported a significantly lower serum AP level in the 40 mg/kg/day caffeine group than in the controls (437.30 ± 88.84 U/L vs. 713.75 ± 98.73 U/L, *p* < 0.05) [25]. However, Naidoo and Islam [25] reported an insignificantly lower serum AP level in the 20 mg/kg caffeine group than in the control group. Two studies measured the serum LDH level, of which 1 study [24] reported a significantly lower serum LDH level (233 ± 35 U/L vs. 458 ± 31 U/L, *p* < 0.05) [24] and the other study [25] reported an insignificantly lower serum LDH level in the caffeine group than in the control group. Two studies reported serum albumin, of which the 30 mg/kg/day caffeine group in the study of Helal, Ayoub [28] had a significantly higher serum albumin level (3.42 + 0.09 g/dL vs. 3.05 + 0.07 g/dL, *p* < 0.05) [28]. Helal, Ayoub [28] and the 20 mg/kg/day caffeine group had an insignificantly higher serum albumin level than the controls. In addition, 1 study [24] reported an insignificantly lower serum albumin level in the caffeine group than in the control group. Two studies measured the serum total bilirubin, and both [24, 28] reporting a significantly lower serum total bilirubin in the caffeine group than in the control group (1.6 ± 0.2 µmol/L vs. 2.4 ± 0.1 µmol/L [24] and 0.4 + 0.037 mg/L [caffeine 20 mg/kg/day group] [28] and 0.4 ± 0.04 mg/L [caffeine 30 mg/kg/day group] vs. 0.66 ± 0.06 mg/L [28], *p* < 0.05 for all).

#### Effect of caffeine vs. control on insulin resistance

Nine studies measured the serum fasting glucose: 4 studies [8, 20, 24, 25], the HSDM arm [4], and the 50 and 100 mg/kg/day caffeine group of Kagami, Morita [23] reported a significantly lower serum fasting glucose level in the caffeine group than in the control group (most significantly, 156 ± 8 mg/dL vs. 205 ± 11 mg/dL [20] and 107.8 ± 1.9 mg/dL vs. 259.5 ± 33.1 mg/dL; *p* < 0.001 for both) [8]; however, 2 studies [19, 27] reported an insignificantly higher serum fasting glucose level in the caffeine group than in the control group, whereas 1 study [21], the HFDM arm [4], and the 10 mg/kg/day caffeine group of Kagami, Morita [23] reported an insignificantly lower serum fasting glucose level in the caffeine group than in the control group. Of the 2 studies that measured the serum postprandial glucose level, 1 study [19] reported a significantly lower level (283.3 ± 19.6 mg/dL vs. 373 ± 19.4 mg/dL, *p* < 0.05) [19] and the other [25] reported an insignificantly lower serum postprandial glucose level in the caffeine group than in the control group. Three studies measured the area under the glucose curve: 2 studies [22, 24] reported a significantly lower area under the glucose curve in the caffeine group than in the control group (area under the glucose curve figure of Tofovic, Salah [22], *p* < 0.05) and (562 ± 14 mmol/L/min vs. 771 ± 10 mmol/L/min, *p* < 0.05) [24] and 1 study [19] reported an insignificantly lower area under the glucose curve in the caffeine group than in the control group. Eight studies measured the serum fasting insulin level, of which 1 study [25] reported a significantly higher serum fasting insulin level in the caffeine group than in the control group (25.81 ± 5.57 pmol/L [caffeine 20 mg/kg/day group] [25] and 21.53 ± 2.91 pmol/L [caffeine 40 mg/kg/day group] vs. 9.16 ± 1.64 pmol/L, *p* < 0.05) [25]; however, 3 studies [8, 19, 20] and the HFDM arm [4] reported a significantly lower serum fasting insulin level in the caffeine group than in the control group (most significantly, 406.8 ± 82.3 pg/mL vs. 1176.4 ± 157.4 pg/mL [8], 69.9 ± 9.4 µU/mL vs. 88.2 ± 6.0 µU/mL [20], and 1.84 ± 0.53 mg/L vs. 5.48 ± 0.22 mg/L [4],, *p* < 0.001 for all). Furthermore, 1 study [24] and the 50 mg/kg/day caffeine group of Kagami, Morita [23] reported an insignificantly higher, and 2 studies [21, 22], the HSDM arm [4], and the 10 and 100 mg/kg/day caffeine groups of Kagami, Morita [23] reported an insignificantly lower, serum fasting insulin level in the caffeine group than in the control group. One study [19] measured serum postprandial insulin level and reported a significantly lower serum postprandial insulin level in the caffeine group than in the control group (110.6 ± 3.4 µIU/mL vs. 146.3 ± 8.5 µIU/mL, *p* < 0.05) [19]. One study [19] that measured the area under the insulin curve and reported a significantly lower area under the insulin curve (198.0 ± 5.9 µIU/mL × h vs. 257.77 ± 12.9 µIU/mL × h, *p* < 0.05) [19]. Nine studies measured the fluid intake, and 3 among those studies [19, 20, 22] reported a significantly lower fluid intake in the caffeine group than in the control group (most significantly, 86 ± 5 mL/kg/d vs. 111 ± 8 mL/kg/d, *p* < 0.001) [20]. Nonetheless, 3 studies [18, 21, 24] and the HFDM [4] reported an insignificantly higher fluid intake in the caffeine group than in the control group; and 2 studies [14, 25] and the HSDM arm [4] reported an insignificantly lower fluid intake in the caffeine group than in the control group. Five studies measured the urinary volume, and 1 study [8] reported a significantly higher urinary volume in the caffeine group than in the control group (Suzuki, Shindo [8] abstract), whereas 4 studies [14, 19, 20, 22] reported a significantly lower urinary volume in the caffeine group than in the control group (most significantly, 20.4 ± 3.9 mL/min/g of kidney vs. 33.5 ± 3.7 mL/min/g, *p* < 0.001) [20]. Of the three studies that measured urinary glucose, all [19, 20, 22] reported a significantly lower urinary glucose level in the caffeine group than in the control group (most significantly, 1.6 ± 0.4 g/day vs. 2.1 ± 0.5 g/day, *p* < 0.005) [20]. Five studies conducted an OGTT (and all measured plasma glucose levels at 30 and 60 min), of which 4 studies [20, 22, 24, 25] and the 100 mg/kg caffeine group of Kagami, Morita [23] reported a significantly lower initial plasma glucose level in the caffeine group than in the control group (initial plasma glucose level in the Figure [20, 22, 23, 25], *p* < 0.05; 4.1 ± 0.2 mmol/L vs. 5.0 ± 0.1 mmol/L, *p* < 0.05) [24]; however, the 10 and 50 mg/kg caffeine group of Kagami, Morita [23] reported an insignificantly lower initial plasma glucose level in the caffeine group than in the control group. At 30 min after the OGTT, the 50 and 100 mg/kg/day caffeine groups of Kagami, Morita [23] reported a significantly lower plasma glucose level (figure, *p* < 0.01 [23] and figure, *p* < 0.001 [23], respectively), 1 study [20] reported an insignificantly higher plasma glucose level, and 3 studies [22, 24, 25] and the 10 mg/kg/day caffeine group of Kagami, Morita [23] reported an insignificantly lower plasma glucose level in the caffeine group than in the control group. At 60 min after the OGTT, 1 study [23] reported a significantly lower plasma glucose level at 60 min (figure, in the 10 mg/kg caffeine group, *p* < 0.05 [23] and figure, in the 50 and100 mg/kg caffeine groups, *p* < 0.001) [23] whereas 4 studies [20, 22, 24, 25] reported an insignificantly lower plasma glucose level in the caffeine group than in the control group. At 90 min, the 20 and 40 mg/kg/day caffeine groups [25] of Naidoo and Islam [25] reported a significantly (*p* < 0.05) [25] and insignificantly lower plasma glucose level, respectively, Naidoo and Islam [25] in the caffeine group than in the control group. Among the 4 studies that measured the plasma glucose level at 120 min in the OGTT, 3 studies [20, 22, 24] and the 20 mg/kg/day caffeine group of Naidoo and Islam [25] reported a significantly lower plasma glucose level (figure, in [20, 22, 24] and the 20 mg/kg caffeine group of Naidoo and Islam [25], *p* < 0.05) and the 40 mg/kg caffeine group of Naidoo and Islam [25] reported an insignificantly lower plasma glucose level in the caffeine group than in the control group. The study [24] that involved an ITT reported an insignificantly lower ITT level initially, at 30 min and at 60 min, but a significantly lower ITT level at 120 min, in the caffeine group than in the control group (figure, *p* < 0.05) [24].

#### Effect of caffeine vs. control on hypertension

Seven studies measured the SBP, among which 1 [20] reported a significantly higher SBP (figure of Tofovic, Kost [20], *p* < 0.05), 3 [8, 16, 24] reported a significantly lower SBP (most significantly, SBP figure of Choi, Lee [16] and 141.9 ± 2.7 mmHg vs. 154.2 ± 2.8 mmHg, both *p* < 0.01) [8], 1 [19] reported an insignificantly higher SBP, and 2 [14, 17] reported an insignificantly lower SBP in the caffeine group than in the control group. Among the 4 studies that measured DBP, 1 [20] reported a significantly higher DBP (figure of Tofovic, Kost [20], *p* < 0.05), 1 [8] reported a significantly lower DBP (94.2 ± 5.3 mmHg vs. 110.1 ± 3.1 mmHg, *p* < 0.05) [8], 1 [19] reported an insignificantly higher DBP, and 1 [17] reported an insignificantly lower DBP in the caffeine group than in the control group. Of the 6 studies that measured the MAP, 2 [18, 20] reported a significantly higher MAP (124 ± 7 mmHg in the 0.1 mg/mL caffeine group vs. 117 ± 10 mmHg, *p* < 0.05 [18]; 139 ± 16 mmHg in the 0.2 mg/mL caffeine group vs. 117 ± 10 mmHg, *p* < 0.001 [18]; and figure of Tofovic, Kost [20], *p* < 0.05) and the HFDM [4] and HSDM [4] arms reported a significantly lower MAP in the caffeine group than in the control group (86.92 ± 3.96 mmHg vs. 108.04 ± 5.30 mmHg, *p* < 0.01 in the HFDM [4] and 92.13 ± 1.82 mmHg vs. 104.69 ± 2.72 mmHg, *p* < 0.05 in the HSDM) [4]; however, 2 studies [19, 22] reported an insignificantly higher MAP and 1 study [17] reported an insignificantly lower MAP in the caffeine group than in the control group.

For a summary of the results mentioned in this section, refer to Table 2. For detailed results refer to the Supplementary Data.

**Table 2:** Summary results of the effect of caffeine in the interventional group compared with the control group

Outcome	Outcome Measure	Significantly Higher	Significantly Lower	Insignificantly Higher	Insignificantly Lower	Studies Reporting the Outcome Measure, N
**Obesity**	Food Intake	-	5 studies [15, 19, 20, 22, 29]	2 studies [8, 24] + HSDM [4]	2 studies [14, 25] + HFDM [4]	10
	Energy Intake	-	1 study [29]	1 study [24]	1 study [21]	3
	Final Weight	1 study [18]	8 studies [8, 19–22, 24, 26, 29]	1 study [25]	1 study [23]	11
	Change in Body Weight	1 study [27]	1 study [15] + HFDM [4]	-	HSDM [4]	3
	Body Fat Percentage	-	2 studies [26, 29]	-	-	2
	Whole-body Fat Weight	-	2 studies [24, 26]	-	-	2
	Whole-body White Adipose Tissue Weight	-	1 study [29]	-	-	1
	Body Fat-pad Weight	-	3 studies [21, 24, 29] + HFDM [4]	-	HSDM [4]	4
**Dyslipidemia**	Serum Triglycerides	1 study [24]	3 studies [26, 28, 29]	-	5 studies [15, 19, 20, 22, 25]	9
	Serum Total Cholesterol	5 studies [15, 19, 20, 22, 24]	2 studies [26, 28]	-	3 studies [25, 27, 29]	10
	LDL-C	-	3 studies [25, 26, 29]	1 study [27]	-	4
	HDL-C	2 studies [15, 28]	-	1 study [29]	3 studies [25–27]	6
	Serum Non-esterified Fatty Acids	1 study [24]	2 studies [8, 29] + HSDM [4]	-	HFDM [4]	4
**Hepatic Steatosis**	Liver Triglycerides	-	1 study [15]	-	-	1
	Liver Cholesterol	-	-	1 study [15]	-	1
	Liver Weight	-	-	2 studies [15, 24] + Naidoo and Islam [25]	Naidoo and Islam [25]	3
**Hepatic Dysfunction**	Serum AST	-	2 studies [24, 28]	-	3 studies [25, 27, 29]	5
	Serum ALT	-	3 studies [24, 28, 29]	1 study [27]	1 study [25]	5
	Serum AP	1 study [24]	Naidoo and Islam [25]	-	Naidoo and Islam [25]	2
	Serum LDH	-	1 study [24]	-	1 study [25]	2
	Serum Albumin	Helal, Ayoub [28]	-	Helal, Ayoub [28]	1 study [24]	2
	Serum Total Bilirubin	-	2 studies [24, 28]	-	-	2
**Insulin Resistance**	Serum Fasting Glucose	-	4 studies [8, 20, 24, 25] + HSDM [4] + Kagami, Morita [23]	2 studies [22, 27]	1 study [21] + HFDM [4] + Kagami, Morita [23]	9
	Serum Postprandial Glucose	-	1 study [19]	-	1 study [25]	2
	Area Under the Glucose Curve	-	2 studies [22, 24]	-	1 study [19]	3
	Serum Fasting Insulin	1 study [25]	3 studies [8, 19, 20] + HFDM [4]	1 study [24] + Kagami, Morita [23]	2 studies [21, 22] + HSDM [4] + Kagami, Morita [23]	8
	Serum Postprandial Insulin	-	1 study [19]	-	-	1
	Area Under the Insulin Curve	-	1 study [19]	-	-	1
	Fluid Intake	-	3 studies [19, 20, 22]	3 studies [18, 21, 24] + HFDM [4]	2 studies [14, 25] + HSDM [4]	9
	Urinary Volume	1 study [8]	4 studies [14, 19, 20, 22]	-	-	5
	Urinary Glucose	-	3 studies [19, 20, 22]	-	-	3
	OGTT Initial	-	4 studies [20, 22, 24, 25] + Kagami, Morita [23]	-	Kagami, Morita [23]	5
	OGTT-30 min	-	Kagami, Morita [23]	1 study [20]	3 studies [22, 24, 25] + Kagami, Morita [23]	5
	OGTT-60 min	-	1 study [23]	-	4 studies [20, 22, 24, 25]	5
	OGTT-90 min	-	Naidoo and Islam [25]	-	Naidoo and Islam [25]	1
	OGTT-120 min	-	3 studies [20, 22, 24] + Naidoo and Islam [25]	-	Naidoo and Islam [25]	4
	ITT Initial	-	-	-	1 study [24]	1
	ITT-30 min	-	-	-	1 study [24]	1
	ITT-60 min	-	-	-	1 study [24]	1
	ITT-120 min	-	1 study [24]	-	-	1
**Hypertension**	Systolic Blood Pressure	1 study [20]	3 studies [8, 16, 24]	1 study [19]	2 studies [14, 17]	7
	Diastolic Blood Pressure	1 study [20]	1 study [8]	1 study [19]	1 study [17]	4
	Mean Arterial Blood pressure	2 studies [18, 20]	HFDM [4] + HSDM [4]	2 studies [19, 22]	1 study [17]	6

*AST* aspartate transaminase, *ALT* alanine transaminase, *AP* alkaline phosphatase, *HDL-C* high density lipoprotein cholesterol, *HFDM* high-fat diet model, *HSDM* high-sucrose diet model, *ITT* insulin tolerance test, *LDH* lactate dehydrogenase, *LDL-C* low density lipoprotein cholesterol, *OGTT* oral glucose tolerance test

## Discussion

To the best of our knowledge, this systematic review constitutes the only review that focused solely on the in vivo effect of caffeine on the cardiometabolic markers of the metabolic syndrome in the rat model. In this systematic review, caffeine was found to lower food intake in rats, but with an inconclusive significance [4, 14, 15, 19, 20, 22, 25, 29]. Energy intake was lower in animals that received caffeine therapy; however, the significance of this effect remains unclear [21, 29]. The reduction in food intake and energy intake supports the therapeutic potential of caffeine in satiety promotion and appetite reduction, which warrants further experimental research to explore the significance of this effect and its potential therapeutic role in the management of obesity. Most of the studies showed that caffeine significantly lowered the final body weight at the end of the experiment, thereby yielding favorable results in animal models of the metabolic syndrome [8, 19–22, 24, 26, 29]. The change in body weight gain from the baseline was lower in the caffeine therapy group, but the significance was indeterminate [4, 15]. The body fat percentage was significantly lower in caffeine-treated animals than in controls [26, 29]. Caffeine significantly lowered the whole-body fat weight in the experimental subjects compared with that in their counterparts [24, 26]. The whole-body white adipose tissue weight was significantly lower following caffeine therapy but was only reported in one study and requires further confirmatory research [29]. The body fat-pad weight was significantly lower in rats treated with caffeine than in controls [4, 21, 24, 29]. All obesity related outcome measures in our study point to a weight reducing desirable effect of caffeine, which is possibly mediated through satiety promotion and the induction of lipolysis. The lipolysis induced by caffeine can be possibly attributed to the direct effect of caffeine on the adipose tissues in the body or through the caloric deficit caused by caffeine’s satiety promoting effect. Therefore, we recommend that future research explores the use of caffeine as adjunct pharmacological agent in the management of obesity or metabolic syndrome.

In terms of dyslipidemia, caffeine lowered serum triglyceride levels in subjects; however, there is no clear consensus on the significance of this effect [15, 19, 20, 22, 25, 26, 28, 29]. There is no clear conclusive consensus on the effect of caffeine on serum total cholesterol. However, most studies with significant results might indicate a caffeine-induced increase in the serum total cholesterol level which could be due to the increase in serum HDL-C as cholesterol gets mobilized from blood vessels towards the liver [15, 19, 20, 22, 24]. In contrast, caffeine significantly lowered serum LDL-C levels, which has a favorable effect on the dyslipidemic component of the metabolic syndrome, which furthermore supports the decrease in cholesterol transport from the liver towards the blood vessels [25, 26, 29]. Caffeine’s effect on HDL-C was inconclusive and there was no clear consensus; however, most studies with significant results found a favorable increase in the serum HDL-C level suggesting a shift in cholesterol transport from blood vessels towards the direction of the liver [15, 28]. Serum non-esterified fatty acids were significantly lower in animals treated with caffeine therapy than in controls which supports caffeine’s role in decreasing lipids in the serum of dyslipidemic subjects [4, 8, 29]. All dyslipidemia outcome measures in our systematic review suggest an active role of caffeine in decreasing the amount of lipids in the serum whilst shifting the direction of cholesterol transport towards the liver. The shift in cholesterol transport supports the anti-atherosclerotic effect of caffeine and its potential in reducing the size of atherosclerotic plaques. Therefore, we recommend that future research focuses on determining the significance of the anti-atherosclerotic effects of caffeine in the setting of metabolic syndrome and its potential as an adjunct therapy in treating the resultant dyslipidemia and atherosclerosis as compared to the current standard of care.

In terms of insulin resistance, the OGTT, glucose levels were initially significantly lower in caffeine-treated animals than in controls [20, 22–25], but this effect at 30 [22–25] and 60 min was nonsignificant [20, 22, 24, 25] and was indeterminate at 90 min [25]. However, at 120 min, glucose levels were significantly lower in caffeine-treated rats, which indicated better glycemic control due to a decrease in insulin resistance which exerts a favorable effect on the diabetes-related component of the metabolic syndrome [20, 22, 24, 25]. Serum fasting insulin levels decreased with caffeine therapy, but there is no consensus on the significance of this effect [4, 8, 19–23]. Only one study reported on serum postprandial insulin and the area under insulin curve, both of which were significantly reduced; however, further confirmatory research is required [19]. The values on the ITT were insignificantly lower initially, at 30 min, and at 60 min, and the significance remains inconclusive as this value was reported by only one study [24]. However, the result on the ITT at 120 min was significantly lower in caffeine-treated subjects, which exerts a positive effect on the diabetes-related component of the metabolic syndrome; however, this effect was reported by only one study [24]. Our review suggests that caffeine decreases insulin resistance in subjects with metabolic syndrome as observed in the OGTT glucose levels which is supported by the decrease in the state of hyperinsulinemia reported in the serum fasting insulin levels, serum postprandial insulin levels, area under insulin curve and ITT. The decrease in insulin resistance observed in our review due to caffeine intake in the metabolic syndrome subjects could be attributed to the decrease in the amount of adipose tissue and possibly the attenuation of the liver’s gluconeogenesis. Serum fasting glucose levels were significantly decreased with caffeine consumption, thereby yielding favorable results in the metabolic syndrome rat model [4, 8, 20, 23–25]. Serum postprandial glucose levels decreased following caffeine therapy, but the significance is yet to be determined [19, 25]. The area under the glucose curve was lower in caffeine-treated subjects, but with indeterminate significance [19, 22, 24]. The decrease in serum fasting glucose, serum post prandial glucose and the area under the glucose curve all confirm the improvement of glycemic control as a result of decreased insulin resistance coupled with a decreased in hyperinsulinemia. Fluid intake decreased in the caffeine-treated group, but this effect had no clear significance [4, 14, 19, 20, 22, 25]. Urinary volume was significantly reduced with caffeine therapy, which suggests a significant decrease in fluid intake due to caffeine consumption [14, 19, 20, 22]. Urinary glucose was significantly decreased due to caffeine intake and yielded favorable results in terms of the diabetes-related component of the metabolic syndrome [19, 20, 22]. Furthermore, the improvement in glycemic control due to caffeine was reflected in clinically significant outcomes that are affected by the diabetic component of the metabolic syndrome such as fluid intake, urinary volume and urinary glucose. Therefore, we recommend further experimental research into the therapeutic use of caffeine as adjunct therapy in the management of the diabetic component of metabolic syndrome as compared to the current standard of care.

In terms of hepatic steatosis and hepatic dysfunction, only one study reported on liver triglycerides and found that triglycerides were significantly lower in caffeine-treated groups than in controls; however, this effect needs to be confirmed in further research [15]. Liver cholesterol levels, on the other hand, were found to be insignificantly higher, which may support the effects observed in our dyslipidemia outcome measures which suggested a shift in cholesterol transport from the blood vessels towards the liver, however this outcome needs further research as only one study measured it [15]. There was no clear consensus on the effect of caffeine on the liver weight as all studies that reported this effect had nonsignificant results [15, 24, 25]. Serum AST levels were lower in caffeine-treated subjects, although the significance is yet to be clearly determined [24, 25, 27–29]. Serum ALT levels were significantly lowered by caffeine therapy [24, 28, 29]. The effect of caffeine on serum AP is unclear and requires further research [24, 25]. Serum LDH levels decreased with caffeine treatment but the significance of this finding is indeterminate [24, 25]. Caffeine’s effect on serum albumin is unclear and requires further research as there is no consensus [24, 28]. Serum total bilirubin levels were significantly decreased due to caffeine therapy [24, 28]. Our outcome measures related to hepatic steatosis and dysfunction indicate that caffeine has a positive hepatoprotective effects such as reducing liver enzymes and optimizing the liver’s metabolic activity. Our systematic review suggests that caffeine optimized the hepatic function in the setting of metabolic syndrome through increasing cholesterol uptake from the serum, decreasing serum total bilirubin and potentially attenuating gluconeogenesis through the decrease in insulin resistance. The optimization of the hepatic function by caffeine resulted in the attenuation of dyslipidemia, hepatic dysfunction and hyperglycemia, respectively. Therefore, we recommend further research on the caffeine’s effect on hepatic steatosis and dysfunction in the setting of metabolic syndrome to assess the significance of its role as a potential optimizing hepatic agent in the management of metabolic syndrome and hepatic steatosis.

SBP was lower in the caffeine-treated group than in controls, but there is no clear consensus on the significance [8, 14, 16, 17, 24]. The effect of caffeine on DBP was inconclusive due to a lack of consensus in reporting this effect [8, 17, 19, 20]. MAP was higher with caffeine therapy although the significance is inconclusive as there is no consensus [18–20, 22].

The evidence included in the review had some key limitations, such as the variability of the metabolic syndrome models that were utilized, which induced some substantial challenges during the synthesis process and may have resulted in a lack of consensus on the significance of the findings. Another very important limitation is the experimental duration of the studies which varied greatly and might have disproportionately influenced the outcome measures of the review. Furthermore, a significant limitation of this review was the inclusion of studies that were only published in the English language due to the authors language barrier limitations, which might have resulted in the exclusion of studies written in different languages that were relevant to this review. The heterogeneity in the methods used to assess the desired outcome measures and the difference in units used greatly hampered any attempt at pursuing a meta-analysis. A major limitation of the studies included was the reporting of outcome measures in the form of graphs without any mention of the exact mean and standard deviation in the results section, thereby rendering efforts at conducting a meta-analysis futile. This resulted in resorting to a qualitative synthesis rather than a quantitative synthesis. The lack of a quantitative synthesis in our review might have resulted in the under characterization of caffeine’s effect on the metabolic syndrome and its potential as a therapeutic agent in human subjects. Although, at the time of writing of this review, a substantial amount of time has passed from the end point of the literature search, the large number of outcome measures that would need to be extracted and resynthesized from newly included studies of an updated endpoint, would have required a considerable amount of time, leading to the reoccurrence of same limitation. Therefore, the authors deemed it infeasible to update the literature search endpoint.

This review represents a stepping stone to the exploration of caffeine’s metabolic effects in the setting of the metabolic syndrome, especially as caffeine is one of the most used substances worldwide and has the potential to be used clinically as adjuvant therapy. Caffeine’s desirable effects of decreasing obesity, reducing dyslipidemia, improving glycemic control and optimizing liver function could potentially be used clinically in treating patients with morbid obesity, diabetes, dyslipidemia, hepatic steatosis and the metabolic syndrome. Therefore, we recommend that future experimental rat model studies use a more homogenous framework, models, and outcome measures to limit the amount of potential confounding factors that might impact the synthesis of future reviews. Moreover, we recommend that future research should focus on caffeine’s effect on dyslipidemia, hepatic steatosis, hepatic dysfunction, and hypertension in the setting of the metabolic syndrome in rat models. Furthermore, we believe that more research needs to be conducted to support the existing evidence in the literature on the effects of caffeine on obesity and insulin resistance before any human clinical trials are considered.

## Conclusion

We found that caffeine has favorable effects on the metabolic syndrome in the rat model, chiefly on the insulin resistance and obesity components. We encourage future research on caffeine's effect on dyslipidemia, hepatic steatosis, hepatic dysfunction, and hypertension, as this is essential for caffeine to be used as a novel adjuvant therapy for the metabolic syndrome. In addition, we recommend conducting further research that supports the evidence on caffeine’s effect on obesity and insulin resistance before consideration of any human clinical trial.

## Supplementary Information

**Additional file 1.**

## Data Availability

All data generated or analyzed during this study are included in this published article and its supplementary information files.
