# Supplementary material for: Systematic review of the effect of caffeine therapy effect on cardiometabolic markers in rat models of the metabolic syndrome
Source: BMC Endocr Disord. 2023 Feb 6;23:34. doi: 10.1186/s12902-023-01288-4 (PMC9901135; doi:10.1186/s12902-023-01288-4)
Supplement: Supplementary file 1 — Additional file 1. [file 12902_2023_1288_MOESM1_ESM.docx]

Supplementary Material

Isa Abdulla Alhadi^1^, Ahmed Mohammed Al-Ansari^1^, Aseel AlSaleh^1^ and Ahmed M. Abdulla Alabbasi^1^*

*Correspondence concerning this article should be addressed to Ahmed M. Abdulla Alabbasi, PhD, Department of Gifted Education, Arabian Gulf University, P.O. Box: 26671, Manama, Bahrain. Telephone (+973)17239414, Fax (+973)17239696. Email: ahmedmda@agu.edu.bh

^1^ Arabian Gulf University

# Supplementary Data

The detailed search strategy is found at the supplementary data. Detailed tables containing the diets, age and sex of rats in each study are also provided. Furthermore, detailed results with extracted information of the results are also provided.

**Search strategy:**

**PubMed:** ((caffeine[tw] OR 1,3,7-Trimethylxanthine[tw] OR Vivarin[tw] OR Caffedrine[tw] OR Coffeinum N[tw] OR Dexitac[tw] OR No Doz[tw] OR Quick-Pep[tw] OR Quick Pep[tw] OR QuickPep[tw] OR Coffeinum Purrum[tw]) AND (metabolic syndrome[tw] OR Metabolic Syndromes[tw] OR Syndrome, Metabolic[tw] OR Syndromes, Metabolic[tw] OR Metabolic Syndrome X[tw] OR Insulin Resistance Syndrome X[tw] OR Syndrome X, Metabolic[tw] OR Syndrome X, Insulin Resistance[tw] OR Metabolic X Syndrome Syndrome, Metabolic X[tw] OR X Syndrome, Metabolic[tw] OR Dysmetabolic Syndrome X[tw] OR Syndrome X, Dysmetabolic[tw] OR Reaven Syndrome X[tw] OR Syndrome X, Reaven[tw] OR Cardiovascular Syndrome[tw] OR Cardiovascular Syndrome, Metabolic[tw] OR Cardiovascular Syndromes, Metabolic[tw] OR Syndrome, Metabolic Cardiovascular[tw]) AND (Rat[tw] OR Rattus[tw] OR Rattus norvegicus[tw] OR Rats, Norway[tw] OR Rats, Laboratory[tw] OR Laboratory Rat[tw] OR Laboratory Rats[tw] OR Rat, Laboratory[tw] OR Mus[tw] OR Mouse[tw] OR Mus musculus[tw] OR Mice, House[tw] OR House Mice[tw] OR Mouse, House[tw] OR House Mouse[tw] OR Mus domesticus[tw] OR Mus musculus domesticus domesticus, Mus musculus[tw] OR Mice, Laboratory[tw] OR Laboratory Mice[tw] OR Mouse, Laboratory[tw] OR Laboratory Mouse[tw] OR Mouse, Swiss[tw] OR Swiss Mouse[tw] OR Swiss Mice[tw] OR Mice, Swiss[tw])) OR ((caffeine[tw] OR 1,3,7-Trimethylxanthine[tw] OR Vivarin[tw] OR Caffedrine[tw] OR Coffeinum N[tw] OR Dexitac[tw] OR No Doz[tw] OR Quick-Pep[tw] OR Quick Pep[tw] OR QuickPep[tw] OR Coffeinum Purrum[tw]) AND (metabolic syndrome[tw] OR Metabolic Syndromes[tw] OR Syndrome, Metabolic[tw] OR Syndromes, Metabolic[tw] OR Metabolic Syndrome X[tw] OR Insulin Resistance Syndrome X[tw] OR Syndrome X, Metabolic[tw] OR Syndrome X, Insulin Resistance[tw] OR Metabolic X Syndrome Syndrome, Metabolic X[tw] OR X Syndrome, Metabolic[tw] OR Dysmetabolic Syndrome X[tw] OR Syndrome X, Dysmetabolic[tw] OR Reaven Syndrome X[tw] OR Syndrome X, Reaven[tw] OR Cardiovascular Syndrome[tw] OR Cardiovascular Syndrome, Metabolic[tw] OR Cardiovascular Syndromes, Metabolic[tw] OR Atherosclerosis[tw] OR Athero-sclerosis[tw] OR dyslipidemia[tw] OR hyperlipidemia[tw] OR Atheroscleroses[tw] OR atherogenesis[tw] OR Dyslipidemias[tw] OR Dyslipoproteinemias[tw] OR Dyslipoproteinemia[tw] OR Hyperlipidemias[tw] OR Lipidemia[tw] OR Lipidemias[tw] OR Lipemia[tw] OR Lipemias[tw] OR Diabetes Mellitus, Type II[tw] OR Diabetes Mellitus, Noninsulin-Dependent[tw] OR Diabetes Mellitus, Ketosis-Resistant[tw] OR Diabetes Mellitus, Ketosis Resistant[tw] OR Ketosis-Resistant Diabetes Mellitus[tw] OR Diabetes Mellitus, Non Insulin Dependent[tw] Diabetes Mellitus, Non-Insulin-Dependent[tw] OR Non-Insulin-Dependent Diabetes Mellitus[tw] OR Diabetes Mellitus, Stable[tw] OR Stable Diabetes Mellitus[tw] OR Diabetes Mellitus, Type II[tw] OR NIDDM[tw] OR Diabetes Mellitus, Noninsulin Dependent[tw] OR Diabetes Mellitus, Maturity-Onset[tw] OR Diabetes Mellitus, Maturity Onset[tw] OR Maturity-Onset Diabetes Mellitus[tw] OR Maturity Onset Diabetes Mellitus[tw] OR MODY[tw] OR Diabetes Mellitus, Slow-Onset[tw] OR Diabetes Mellitus, Slow Onset[tw] OR Slow-Onset Diabetes Mellitus[tw] OR Type 2 Diabetes Mellitus[tw] OR Noninsulin-Dependent Diabetes Mellitus[tw] OR Noninsulin Dependent Diabetes Mellitus[tw] OR Maturity-Onset Diabetes[tw] OR Diabetes, Maturity-Onset[tw] OR Maturity Onset Diabetes[tw] OR Type 2 Diabetes[tw] OR Diabetes, Type 2[tw] OR Diabetes Mellitus, Adult-Onset[tw] OR Adult-Onset Diabetes Mellitus[tw] OR Diabetes Mellitus, Adult Onset[tw] OR Non-alcoholic Fatty Liver Disease[tw] OR Non alcoholic Fatty Liver Disease[tw] OR NAFLD[tw] OR Nonalcoholic Fatty Liver Disease[tw] OR Fatty Liver, Nonalcoholic[tw] OR Fatty Livers, Nonalcoholic[tw] OR Liver, Nonalcoholic Fatty[tw] OR Livers, Nonalcoholic Fatty[tw] OR Nonalcoholic Fatty Liver[tw] OR Nonalcoholic Fatty Livers[tw] OR Nonalcoholic Steatohepatitis[tw] OR Nonalcoholic Steatohepatitides[tw] OR Steatohepatitides, Nonalcoholic[tw] OR Steatohepatitis, Nonalcoholic[tw] OR Syndrome, Metabolic Cardiovascular[tw]) AND (Rat[tw] OR Rattus[tw] OR Rattus norvegicus[tw] OR Rats, Norway[tw] OR Rats, Laboratory[tw] OR Laboratory Rat[tw] OR Laboratory Rats[tw] OR Rat, Laboratory[tw] OR Mus[tw] OR Mouse[tw] OR Mus musculus[tw] OR Mice, House[tw] OR House Mice[tw] OR Mouse, House[tw] OR House Mouse[tw] OR Mus domesticus[tw] OR Mus musculus domesticus domesticus, Mus musculus[tw] OR Mice, Laboratory[tw] OR Laboratory Mice[tw] OR Mouse, Laboratory[tw] OR Laboratory Mouse[tw] OR Mouse, Swiss[tw] OR Swiss Mouse[tw] OR Swiss Mice[tw] OR Mice, Swiss[tw]))

**Scopus:** (TITLE-ABS-KEY(“Caffeine”) OR TITLE-ABS-KEY(“1,3,7-Trimethylxanthine”) OR TITLE-ABS-KEY(“Vivarin”) OR TITLE-ABS-KEY(“Caffedrine”) OR TITLE-ABS-KEY(“Coffeinum N”) OR TITLE-ABS-KEY(“Dexitac”) OR TITLE-ABS-KEY(“No Doz”) OR TITLE-ABS-KEY(“Quick-Pep”) OR TITLE-ABS-KEY(“Quick Pep”) OR TITLE-ABS-KEY(“QuickPep”) OR TITLE-ABS-KEY(“Coffeinum Purrum”)) AND (TITLE-ABS-KEY(“metabolic syndrome”) OR TITLE-ABS-KEY(“Metabolic Syndromes”) OR TITLE-ABS-KEY(“Syndrome, Metabolic”) OR TITLE-ABS-KEY(“Syndromes, Metabolic”) OR TITLE-ABS-KEY(“Metabolic Syndrome X”) OR TITLE-ABS-KEY(“Insulin Resistance Syndrome X”) OR TITLE-ABS-KEY(“Syndrome X, Metabolic”) OR TITLE-ABS-KEY(“Syndrome X, Insulin Resistance”) OR TITLE-ABS-KEY(“Metabolic X Syndrome Syndrome, Metabolic X”) OR TITLE-ABS-KEY(“X Syndrome, Metabolic”) OR TITLE-ABS-KEY(“Dysmetabolic Syndrome X”) OR TITLE-ABS-KEY(“Syndrome X, Dysmetabolic”) OR TITLE-ABS-KEY(“Reaven Syndrome X”) OR TITLE-ABS-KEY(“Syndrome X, Reaven”) OR TITLE-ABS-KEY(“Cardiovascular Syndrome”) OR TITLE-ABS-KEY(“Cardiovascular Syndrome, Metabolic”) OR TITLE-ABS-KEY(“Cardiovascular Syndromes, Metabolic”) OR TITLE-ABS-KEY(“Atherosclerosis”) OR TITLE-ABS-KEY(“Athero-sclerosis”) OR TITLE-ABS-KEY(“dyslipidemia”) OR TITLE-ABS-KEY(“hyperlipidemia”) OR TITLE-ABS-KEY(“Atheroscleroses”) OR TITLE-ABS-KEY(“atherogenesis”) OR TITLE-ABS-KEY(“Dyslipidemias”) OR TITLE-ABS-KEY(“Dyslipoproteinemias”) OR TITLE-ABS-KEY(“Dyslipoproteinemia”) OR TITLE-ABS-KEY(“Hyperlipidemias”) OR TITLE-ABS-KEY(“Lipidemia”) OR TITLE-ABS-KEY(“Lipidemias”) OR TITLE-ABS-KEY(“Lipemia”) OR TITLE-ABS-KEY(“Lipemias”) OR TITLE-ABS-KEY(“Diabetes Mellitus, Type II”) OR TITLE-ABS-KEY(“Diabetes Mellitus, Noninsulin-Dependent”) OR TITLE-ABS-KEY(“Diabetes Mellitus, Ketosis-Resistant”) OR TITLE-ABS-KEY(“Diabetes Mellitus, Ketosis Resistant”) OR TITLE-ABS-KEY(“Ketosis-Resistant Diabetes Mellitus”) OR TITLE-ABS-KEY(“Diabetes Mellitus, Non Insulin Dependent”) OR TITLE-ABS-KEY(“Diabetes Mellitus, Non-Insulin-Dependent”) OR TITLE-ABS-KEY(“Non-Insulin-Dependent Diabetes Mellitus”) OR TITLE-ABS-KEY(“Diabetes Mellitus, Stable”) OR TITLE-ABS-KEY(“Stable Diabetes Mellitus”) OR TITLE-ABS-KEY(“Diabetes Mellitus, Type II”) OR TITLE-ABS-KEY(“NIDDM”) OR TITLE-ABS-KEY(“Diabetes Mellitus, Noninsulin Dependent”) OR TITLE-ABS-KEY(“Diabetes Mellitus, Maturity-Onset”) OR TITLE-ABS-KEY(“Diabetes Mellitus, Maturity Onset”) OR TITLE-ABS-KEY(“Maturity-Onset Diabetes Mellitus”) OR TITLE-ABS-KEY(“Maturity Onset Diabetes Mellitus”) OR TITLE-ABS-KEY(“MODY”) OR TITLE-ABS-KEY(“Diabetes Mellitus, Slow-Onset”) OR TITLE-ABS-KEY(“Diabetes Mellitus, Slow Onset”) OR TITLE-ABS-KEY(“Slow-Onset Diabetes Mellitus”) OR TITLE-ABS-KEY(“Type 2 Diabetes Mellitus”) OR TITLE-ABS-KEY(“Noninsulin-Dependent Diabetes Mellitus”) OR TITLE-ABS-KEY(“Noninsulin Dependent Diabetes Mellitus”) OR TITLE-ABS-KEY(“Maturity-Onset Diabetes”) OR TITLE-ABS-KEY(“Diabetes, Maturity-Onset”) OR TITLE-ABS-KEY(“Maturity Onset Diabetes”) OR TITLE-ABS-KEY(“Type 2 Diabetes”) OR TITLE-ABS-KEY(“Diabetes, Type 2”) OR TITLE-ABS-KEY(“Diabetes Mellitus, Adult-Onset”) OR TITLE-ABS-KEY(“Adult-Onset Diabetes Mellitus”) OR TITLE-ABS-KEY(“Diabetes Mellitus, Adult Onset”) OR TITLE-ABS-KEY(“Non-alcoholic Fatty Liver Disease”) OR TITLE-ABS-KEY(“Non alcoholic Fatty Liver Disease”) OR TITLE-ABS-KEY(“NAFLD”) OR TITLE-ABS-KEY(“Nonalcoholic Fatty Liver Disease”) OR TITLE-ABS-KEY(“Fatty Liver, Nonalcoholic”) OR TITLE-ABS-KEY(“Fatty Livers, Nonalcoholic”) OR TITLE-ABS-KEY(“Liver, Nonalcoholic Fatty”) OR TITLE-ABS-KEY(“Livers, Nonalcoholic Fatty”) OR TITLE-ABS-KEY(“Nonalcoholic Fatty Liver”) OR TITLE-ABS-KEY(“Nonalcoholic Fatty Livers”) OR TITLE-ABS-KEY(“Nonalcoholic Steatohepatitis”) OR TITLE-ABS-KEY(“Nonalcoholic Steatohepatitides”) OR TITLE-ABS-KEY(“Steatohepatitides, Nonalcoholic”) OR TITLE-ABS-KEY(“Steatohepatitis, Nonalcoholic”) OR TITLE-ABS-KEY(“Syndrome, Metabolic Cardiovascular”)) AND (TITLE-ABS-KEY(“Rat”) OR TITLE-ABS-KEY(“Rattus”) OR TITLE-ABS-KEY(“Rattus norvegicus”) OR TITLE-ABS-KEY(“Rats, Norway”) OR TITLE-ABS-KEY(“Rats, Laboratory”) OR TITLE-ABS-KEY(“Laboratory Rat”) OR TITLE-ABS-KEY(“Laboratory Rats”) OR TITLE-ABS-KEY(“Rat, Laboratory”) OR TITLE-ABS-KEY(“Mus”) OR TITLE-ABS-KEY(“Mouse”) OR TITLE-ABS-KEY(“Mus musculus”) OR TITLE-ABS-KEY(“Mice, House”) OR TITLE-ABS-KEY(“House Mice”) OR TITLE-ABS-KEY(“Mouse, House”) OR TITLE-ABS-KEY(“House Mouse”) OR TITLE-ABS-KEY(“Mus domesticus”) OR TITLE-ABS-KEY(“Mus musculus domesticus domesticus, Mus musculus”) OR TITLE-ABS-KEY(“Mice, Laboratory”) OR TITLE-ABS-KEY(“Laboratory Mice”) OR TITLE-ABS-KEY(“Mouse, Laboratory”) OR TITLE-ABS-KEY(“Laboratory Mouse”) OR TITLE-ABS-KEY(“Mouse, Swiss”) OR TITLE-ABS-KEY(“Swiss Mouse”) OR TITLE-ABS-KEY(“Swiss Mice”) OR TITLE-ABS-KEY(“Mice, Swiss”))

**ScienceDirect:** In the Title, abstract, keywords field in advanced search: (“Caffeine” OR “1,3,7-Trimethylxanthine”) AND (“metabolic syndrome” OR “Diabetes mellites” OR “atherosclerosis” OR “dyslipidemia” OR “non alcoholic fatty liver disease”) AND (“mice” OR “rats”)

**Table A:** Sex, Age, and diet in included studies

| Author | Year | Sex | Initial Age (weeks) | Final age (weeks) | Diet | | | |
| --- | --- | --- | --- | --- | --- | --- | --- | --- |
|  |  |  |  |  | **Manufacturer Name / Product Name** | **Macronutrients (%)** | **Ingredients** | **Availability** |
| Ohnishi A Fau - Branch, Branch Ra Fau - Jackson ^14^ | 1986 | - | 5 | 13 | Wayne Lab-Blox (Allied Mills, Inc., Memphis, TN) | - | 173 meq Na+/kg 246 meq K+/kg | ad libitum |
| Sugiyama, Ohishi ^15^ | 1989 | Male | - | - | basal diet (25C) | - | 25% casein, 15% sucrose, 15% Jard~ 2% corn oil, 5% salt mixture,7) 1% vitamin mixture,7) 0.2% choline chloride, 2%cellufosepowder, 2% cholesterol, and a-corn starch | - |
| Choi, Lee ^16^ | 1993 | Male | - | - | - | - | - | - |
| Tofovic and Jackson ^17^ | 1999 | Male | 28 (8 months) | 41 | Wayne Rodent Blox 8604 (sodium, 135 mEq/kg, and potassium 254 mEq/kg; Madison, WI, U.S.A.) | - | sodium,135 mEq/kg and potassium 254 mEq/kg | ad libitum |
| Tanner and Tanner ^18^ | 2001 | Male | 4 | 24 | (Teklad 6% mouse/rat diet 7002; Harlan, Madison, WI) | 24% protein  6% fat | - | ad libitum |
| Tofovic, Kusaka ^19^ | 2001 | Male | 18 | 26 | Pro Lab RMH 3000 rodent diet (PMI Nutrition Inc., St Louis, MO) | - | - | ad libitum |
| Tofovic, Kost ^20^ | 2002 | Male | 8 | 38 | Pro Lab RMH 3000 fastrodent diet (PMI Nutrition Inc., St. Louis, MO, USA) | - | - | ad libitum |
| Park, Jang ^21^ | 2007 | Male | - | - | Modified AIN-93 formulation | 38% carbohydrates 22% protein 40% fats | starch casein shortening | ad libitum |
| Tofovic, Salah ^22^ | 2007 | Male | 11 | 21 | Pro Lab RMH 3000 rodent diet (PMI Nutrition Inc., St. Louis, Missouri, USA) | - | - | ad libitum |
| Kagami, Morita ^23^ | 2008 | Male | 7 | 8 | standard rat chow (CE-2; CLEA Japan Inc., Japan) | - | - | ad libitum |
| Conde, Nunes da Silva ^4^ | 2012 | Male and Female | 12 (3 months) | 15 | Mucedola, Settimo Milanese, Italy | 45% fat  35% carbohydrate 20% protein | - | - |
|  |  |  |  | 16 | sucrose in water (Panlab, Lisbon, Portugal) + standard chow (SDS diets RM1; Probiolo´gica, Sintra, Portugal | standard chow: 7·4% fat 75%carbohydrate 17% protein | 35% sucrose | - |
| Panchal, Wong ^24^ | 2012 | Male | 8 to 9 | 24 to 25 | High carbohydrate, high fat diet plus fructose in water | - | 25% fructose in water 175 g of fructose, 395 g of sweetened condensed milk, 200 g of beef tallow, 155 g of powdered rat food, 25 g of Hubble, Mendel and Wakeman salt mixture, and 50 g of water per kilogram of diet Panchal, Poudyal ^30^ | ad libitum |
| Naidoo and Islam ^25^ | 2014 | Male | 6 | 20 | commercially available rat pellet diet | - | - | ad libitum |
| Xu, Zhang ^26^ | 2015 | Male | - | - | high fat diet | - | 79% of the basal feed, 10% lard, 10% egg yolk powder, 0.5% cholesterol, 0.5% cholate | ad libitum |
| Kumbhar, Une ^27^ | 2016 | Male and Female | 8 to 10 | 14 to 16 | standard diet (Nutrivet Life Sciences, India) | - | - | ad libitum |
| Suzuki, Shindo ^8^ | 2017 | Male | 24 | 29 | standard rat chow (CEII, CLEA, Tokyo, Japan) | - | - | ad libitum |
| Helal, Ayoub ^28^ | 2018 | Male | - | - | high fat diet ICN Pharmaceuticals (NY, USA) | 45% lipid, 30% carbohydrate, and 25% protein | Casein purified high: 20 kg Nitrogen - Corn starch: 39 kg  Corn oil: 3.4 kg Lard: 14.6 kg AIN 76 Mineral Mix 5 kg Alpha cell nonnutritive 16 kg Bulk VDFM (sheet dextrose) 2 kg ^31^ | - |
| Yang, Zhu ^29^ | 2019 | Male | 4 | 15 | High fat diet | 4.32 kcal/g | 78.8% normal diet, 10% lard oil, 10% yolk powder, 1% cholesterol, and 0.2% bile salt. | ad libitum |

**Table B:** Obesity Outcome Measures Results

| Author | Year | Group Name | Obesity Outcome Measures | | | | | | | |
| --- | --- | --- | --- | --- | --- | --- | --- | --- | --- | --- |
|  |  |  | **Food Intake** | **Energy Intake** | **Final Weight** | **Change** | **Body Fat Percentage** | **Whole Body Fat Weight** | **White Adipose Tissue Weight** | **Body Fat Pads** |
|  |  |  |  |  |  |  |  |  |  |  |
| Ohnishi A Fau - Branch, Branch Ra Fau - Jackson ^14^ | 1986 | caffeine | 16 ± 1 (g/d) | - | - | - | - | - | - | - |
|  |  | control | 18 ± 1 (g/d) | - | - | - | - | - | - | - |
| Sugiyama, Ohishi ^15^ | 1989 | caffeine | 152 ± 5* (g/ I4d) | - | - | 60 ± 4* (g/ 14 d) | - | - | - | - |
|  |  | control | 173 ± 3 (g/ I4d) | - | - | 80 ± 3 (g/ 14 d) | - | - | - | - |
| Choi, Lee ^16^ | 1993 | caffeine | - | - | - | - | - | - | - | - |
|  |  | control | - | - | - | - | - | - | - | - |
| Tofovic and Jackson ^17^ | 1999 | caffeine | - | - | - | - | - | - | - | - |
|  |  | control | - | - | - | - | - | - | - | - |
| Tanner and Tanner ^18^ | 2001 | caffeine 0.1 (mg/mL) | - | - | 499 ± 26* (g) | - | - | - | - | - |
|  |  | caffeine 0.2 (mg/mL) | - | - | 508 ± 14** (g) | - | - | - | - | - |
|  |  | control | - | - | 462 ± 29 (g) | - | - | - | - | - |
| Tofovic, Kusaka ^19^ | 2001 | caffeine | 47.4 ± 1.4* (g/kg/day) | - | 614±4.2* (g) | - | - | - | - | - |
|  |  | control | 56.1 ± 2.3 (g/kg/day) | - | 670 ± 14.8 (g) | - | - | - | - | - |
| Tofovic, Kost ^20^ | 2002 | caffeine | 49 ± 3*** (g/kg/day) | - | 650±20*** (g) | - | - | - | - | - |
|  |  | control | 61 ± 1 (g/kg/day) | - | 740 ± 11 (g) | - | - | - | - | - |
| Park, Jang ^21^ | 2007 | caffeine | - | 34.3 ± 4.2 (kJ/d) | 328.4 ± 15.6* (g) | - | - | - | - | epididymal 4.1 ± 0.5* (g) |
|  |  |  |  |  |  |  |  |  |  |  |
|  |  |  |  |  |  |  |  |  |  |  |
|  |  | control | - | 35.6 ± 4.0 (kJ/d) | 392.6 ± 24.2 (g) | - | - | - | - | epididymal 4.9 ± 0.6 (g) |
| Tofovic, Salah ^22^ | 2007 | caffeine | 57 ± 2* (g/kg) | - | 537 ± 7* (g) | - | - | - | - | - |
|  |  | control | 76 ± 2 (g/kg) | - | 605 ± 6 (g) | - | - | - | - | - |
| Kagami, Morita ^23^ | 2008 | caffeine 10 (mg/kg) | - | - | 204 ± 3 (g) | - | - | - | - | - |
|  |  | caffeine 50 (mg/kg) | - | - | 205 ± 4 (g) | - | - | - | - | - |
|  |  | caffeine 100(mg/kg) | - | - | 207 ± 2 (g) | - | - | - | - | - |
|  |  | control | - | - | 209± 3 (g) | - | - | - | - | - |
| Conde, Nunes da Silva ^4^ | 2012 | Caffeine HFDM | 60.58 ± 2.79 (mg/kg/ d) | - | - | 2.39 ± 0.36*** (g/d) | - | - | - | visceral fat: 7.80 ± 0.90*** (g/kg) |
|  |  | Control  HFDM | 62.56 ± 1.99 (mg/kg/d) | - | - | 4.32 ± 0.45 (g/d) | - | - | - | visceral fat: 12.70 ± 0.64 (g/kg) |
|  |  | Caffeine  HSDM | 52.78 ± 3.81 (mg/kg/d) | - | - | 2.51 ± 0.29 (g/d) | - | - | - | visceral fat: 9.91 ± 0.97 (g/kg) |
|  |  | Control  HSDM | 51.22 ± 4.51 (mg/kg/d) | - | - | 2.66 ± 0.25 (g/d) | - | - | - | visceral fat: 10.99 ± 0.86 (g/kg) |
| Panchal, Wong ^24^ | 2012 | caffeine | 23.4 ± 0.4 (g/d) | 492 ± 15 (kJ/d) | 396 ± 6* (g) | - | - | 80 ± 6* (g) | - | Retroperitoneal fat pads: 198 ± 10* (mg/mm tibial length)  Epididymal fat pads: 122 ± 7* (mg/mm tibial length)  Omental fat pads: 83 ± 6* (mg/mm tibial length)  Total abdominal fat pads: 402 ± 21* (mg/mm tibial length) |
|  |  | control | 23.2 ± 0.6 (g/d) | 478 ± 11 (kJ/d) | 480 ± 8 (g) | - | - | 152 ± 7 (g) | - | Retroperitoneal fat pads: 357 ± 21 (mg/mm tibial length)  Epididymal fat pads: 225 ± 13 (mg/mm tibial length)  Omental fat pads: 194 ± 12 (mg/mm tibial length)  Total abdominal fat pads: 775 ± 46 (mg/mm tibial length) |
| Naidoo and Islam ^25^ | 2014 | caffeine 20 (mg/kg) | lower insig | - | higher insig | - | - | - | - | - |
|  |  | caffeine 40 (mg/kg) | lower insig | - | higher insig | - | - | - | - | - |
|  |  | control | - | - | - | - | - | - | - | - |
| Xu, Zhang ^26^ | 2015 | caffeine | - | - | 311.76 ± 46.26* (g) | - | 1.81 ± 0.60* (%) | 5.22 ± 1.81* (g) | - | - |
|  |  | control | - | - | 384.96 ± 32.59 (g) | - | 2.85 ± 0.45 (%) | 10.99 ± 2.24 (g) | - | - |
| Kumbhar, Une ^27^ | 2016 | caffeine | - | - | - | -83 ± 2.32* (g) | - | - | - | - |
|  |  | control | - | - | - | -61 ± 3.31 (g) | - | - | - | - |
| Suzuki, Shindo ^8^ | 2017 | caffeine | higher insig | - | lower sig  (p-value <0.001) | - | - | - | - | - |
|  |  | control | - | - | - | - | - | - | - | - |
| Helal, Ayoub ^28^ | 2018 | caffeine 20 (mg/kg/ day) | - | - | - | - | - | - | - | - |
|  |  | caffeine 30 (mg/kg/ day) | - | - | - | - | - | - | - | - |
|  |  | control | - | - | - | - | - | - | - | - |
| Yang, Zhu ^29^ | 2019 | caffeine | 25.8 ± 2** (g/d) | 111.7 ± 8.9** (kcal/d) | lower sig  (p-value <0.01) | - | 3.07 ± 0.65** (%) | - | 17.3 ± 3.4** (g) | perirenal: 8.4 ± 2.3** (g) epididymal: 8.9 ± 1.5** (g) |
|  |  | control | 28.1 ± 2.1 (g/d) | 121.5 ± 8.9 (kcal/d) | - | - | 4.99 ± 0.47 (%) | - | 29.3 ± 3.3 (g) | perirenal: 16.2 ± 3.6 (g) epididymal: 13.1 ± 2.5 (g) |

Insig = insignificant, sig = significant, kJ = kilo joule; * = p-value < 0.05 vs controls, ** = p-value < 0.01 vs controls, **^ = p-value < 0.005 vs controls, *** = p-value < 0.001 vs controls (higher or lower refers to the caffeine group vs control in that specific measure)

**Table C:** Dyslipidemia Outcome Measures Results

| Author | Year | Group Name | Dyslipidemia Outcome Measures | | | | |
| --- | --- | --- | --- | --- | --- | --- | --- |
|  |  |  | **Total TG** | **Total Cholesterol** | **LDL** | **HDL** | **Serum Non-Esterified Fatty Acids** |
|  |  |  |  |  |  |  |  |
| Ohnishi A Fau - Branch, Branch Ra Fau - Jackson ^14^ | 1986 | caffeine | - | - | - | - | - |
|  |  | control | - | - | - | - | - |
| Sugiyama, Ohishi ^15^ | 1989 | caffeine | 137 ± 13  (mg/100 mL) | 208 ± 13*  (mg/100 mL) | - | 28 ± 2*  (mg/100 mL) | - |
|  |  | control | 144 ± 16  (mg/ 100 mL) | 119 ± 8 (mg/100 mL) | - | 23 ± 1  (mg/100 mL) | - |
| Choi, Lee ^16^ | 1993 | caffeine | - | - | - | - | - |
|  |  | control | - | - | - | - | - |
| Tofovic and Jackson ^17^ | 1999 | caffeine | - | - | - | - | - |
|  |  | control | - | - | - | - | - |
| Tanner and Tanner ^18^ | 2001 | caffeine 0.1 (mg/mL) | - | - | - | - | - |
|  |  | caffeine 0.2 (mg/mL) | - | - | - | - | - |
|  |  | control | - | - | - | - | - |
| Tofovic, Kusaka ^19^ | 2001 | caffeine | lower insig | higher sig (p-value <0.001) | - | - | - |
|  |  | control | - | - | - | - | - |
| Tofovic, Kost ^20^ | 2002 | caffeine | 765 ± 58 (mg/mL) | 628 ± 102**^ (mg/dL) | - | - | - |
|  |  | control | 815 ± 40 (mg/mL) | 225 ± 8 (mg/dL) | - | - | - |
| Park, Jang ^21^ | 2007 | caffeine | - | - | - | - | - |
|  |  |  |  |  |  |  |  |
|  |  |  |  |  |  |  |  |
|  |  | control | - | - | - | - | - |
| Tofovic, Salah ^22^ | 2007 | caffeine | lower insig | higher sig (p-value <0.05) | - | - | - |
|  |  | conrol | - | - | - | - | - |
| Kagami, Morita ^23^ | 2008 | caffeine 10 (mg/kg) | - | - | - | - | - |
|  |  | caffeine 50 (mg/kg) | - | - | - | - | - |
|  |  | caffeine 100(mg/kg) | - | - | - | - | - |
|  |  | control | - | - | - | - | - |
| Conde, Nunes da Silva ^4^ | 2012 | Caffeine  HFDM | - | - | - | - | 391.67 ± 38.23 µM |
|  |  | Control  HFDM | - | - | - | - | 436.45 ± 36.23 µM |
|  |  | Caffeine  HSDM | - | - | - | - | 610.24 ± 41.06 *** µM |
|  |  | Control  HSDM | - | - | - | - | 940.62 ± 89.66 µM |
| Panchal, Wong ^24^ | 2012 | caffeine | 1.5 ± 0.2* (mmol/L) | 2.6 ± 0.2* (mmol/L) | - | - | 5.1 ± 0.4* (mmol/L) |
|  |  | control | 1.0 ± 0.1 (mmol/L) | 2.0 ± 0.2 (mmol/L) | - | - | 2.8 ± 0.3 (mmol/L) |
| Naidoo and Islam ^25^ | 2014 | caffeine 20 (mg/kg) | lower insig | lower insig | lower sig (p-value <0.05) | lower insig | - |
|  |  | caffeine 40 (mg/kg) | lower insig | lower insig | lower sig (p-value <0.05) | lower insig | - |
|  |  | Control | - | - | - | - | - |
| Xu, Zhang ^26^ | 2015 | caffeine | 0.86 ± 0.16* (mmol/L) | 2.78 ± 0.36* (mmol/L) | 0.94 ± 0.18* (mmol/L) | 0.89 ± 0.31 (mmol/L) | - |
|  |  | control | 1.53 ± 0.22 (mmol/L) | 4.32 ± 0.88 (mmol/L) | 3.04 ± 0.93 (mmol/L) | 1.04 ± 0.11 (mmol/L) | - |
| Kumbhar, Une ^27^ | 2016 | caffeine | - | 157 ± 10 (mg/ dL) | 141 ± 9 (mg/dL) | 42 ± 3.9 (mg/dL) | - |
|  |  | control | - | 163 ± 6 (mg/dL) | 133 ± 9 (mg/dL) | 47 ± 4.7 (mg/dL) | - |
| Suzuki, Shindo ^8^ | 2017 | caffeine | - | - | - | - | 0.87 ± 0.04*** (mEq/L) |
|  |  | control | - | - | - | - | 1.88 ± 0.14 (mEq/L) |
| Helal, Ayoub ^28^ | 2018 | caffeine 20 (mg/kg/ day) | 89.6 ± 9* (mg/dL) | 83.6 ± 3* (mg/dL) | - | 52 + 5.1* (mg/dL) | - |
|  |  | caffeine 30 (mg/kg/ day) | 50.6 ± 2.9* (mg/dL) | 81.2 ± 5.8* (mg/dL) | - | 50.1 + 5.1* (mg/dL) | - |
|  |  | control | 131.8 ± 3 (mg/dL) | 110 ± 4.4 (mg/dL) | - | 30.4 + 1.9 (mg/dL) | - |
| . Yang, Zhu ^29^ | 2019 | caffeine | 1.05 ± 0.13** (mmol/L) | 2.55 ± 0.15 (mmol/L) | 0.51 ± 0.05* (mmol/L) | 1.03 ± 0.14 (mmol/L) | 0.31 ± 0.05* (mmol/L) |
|  |  | control | 1.39 ± 0.17 (mmol/L) | 2.84 ± 0.43 (mmol/L) | 0.63 ± 0.07 (mmol/L) | 0.88 ± 0.15 (mmol/L) | 0.39 ± 0.05 (mmol/L) |

Insig = insignificant, sig = significant; * = p-value < 0.05 vs controls, ** = p-value < 0.01 vs controls, **^ = p-value < 0.005 vs controls, *** = p-value < 0.001 vs controls (higher or lower refers to the caffeine group vs control in that specific measure)

**Table D:** Hepatic Steatosis and Dysfunction Outcome Measures Results

| Author | Year | Group Name | Hepatic Steatosis and Dysfunction Outcome Measures | | | | | | | | |
| --- | --- | --- | --- | --- | --- | --- | --- | --- | --- | --- | --- |
|  |  |  | Liver Triglycerides | Liver Cholesterol | AST | ALT | AP | LDH | Serum Albumin | Serum Total Bilirubin | Liver Weight |
|  |  |  |  |  |  |  |  |  |  |  |  |
| Ohnishi A Fau - Branch, Branch Ra Fau - Jackson ^14^ | 1986 | caffeine | - | - | - | - | - | - | - | - | - |
|  |  | control | - | - | - | - | - | - | - | - | - |
| Sugiyama, Ohishi ^15^ | 1989 | caffeine | 40.7 ± 1.4* (mg/g) | 54.8 ± 1.9 (mg/g) | - | - | - | - | - | - | 4.55 ±0.07 (g/100 g body weight) |
|  |  | control | 53.8 ± 2.9 (mg/g) | 51.3 ± 0.9 (mg/g) | - | - | - | - | - | - | 4.46 ±0.08 (g/100 g body weight) |
| Choi, Lee ^16^ | 1993 | caffeine | - | - | - | - | - | - | - | - | - |
|  |  | control | - | - | - | - | - | - | - | - | - |
| Tofovic and Jackson ^17^ | 1999 | caffeine | - | - | - | - | - | - | - | - | - |
|  |  | control | - | - | - | - | - | - | - | - | - |
| Tanner and Tanner ^18^ | 2001 | caffeine 0.1 (mg/mL) | - | - | - | - | - | - | - | - | - |
|  |  | caffeine 0.2 (mg/mL) | - | - | - | - | - | - | - | - | - |
|  |  | control | - | - | - | - | - | - | - | - | - |
| Tofovic, Kusaka ^19^ | 2001 | caffeine | - | - | - | - | - | - | - | - | - |
|  |  | control | - | - | - | - | - | - | - | - | - |
| Tofovic, Kost ^20^ | 2002 | caffeine | - | - | - | - | - | - | - | - | - |
|  |  | control | - | - | - | - | - | - | - | - | - |
| Park, Jang ^21^ | 2007 | caffeine | - | - | - | - | - | - | - | - | - |
|  |  |  |  |  |  |  |  |  |  |  |  |
|  |  |  |  |  |  |  |  |  |  |  |  |
|  |  | control | - | - | - | - | - | - | - | - | - |
| Tofovic, Salah ^22^ | 2007 | caffeine | - | - | - | - | - | - | - | - | - |
|  |  | control | - | - | - | - | - | - | - | - | - |
| Kagami, Morita ^23^ | 2008 | caffeine 10 (mg/kg) | - | - | - | - | - | - | - | - | - |
|  |  | caffeine 50 (mg/kg) | - | - | - | - | - | - | - | - | - |
|  |  | caffeine 100(mg/kg) | - | - | - | - | - | - | - | - | - |
|  |  | control | - | - | - | - | - | - | - | - | - |
| Conde, Nunes da Silva ^4^ | 2012 | Caffeine  HFDM | - | - | - | - | - | - | - | - | - |
|  |  | Control  HFDM | - | - | - | - | - | - | - | - | - |
|  |  | Caffeine  HSDM | - | - | - | - | - | - | - | - | - |
|  |  | Control  HSDM | - | - | - | - | - | - | - | - | - |
| Panchal, Wong ^24^ | 2012 | caffeine | - | - | 80 ± 5* (U/L) | 42 ± 3* (U/L) | 363 ± 20* (U/L) | 233 ± 35* (U/L) | 28.4 ± 0.6 (mg/mL) | 1.6 ± 0.2* (µmol/L) | 331 ± 17 (mg/mm tibial length) |
|  |  | control | - | - | 102 ± 5 (U/L) | 55 ± 3 (U/L) | 261 ± 18 (U/L) | 458 ± 31 (U/L) | 28.6 ± 0.4 (mg/mL) | 2.4 ± 0.1 (µmol/L) | 297 ± 11 (mg/mm tibial length) |
| . Naidoo and Islam ^25^ | 2014 | caffeine 20 (mg/kg) | - | - | 121 ± 29.77 (U/L) | 138.38 ± 33.79 (U/L) | 624 ± 104.03 (U/L) | 471.25 ± 97.10 (U/L) | - | - | 8.94 ± 1.10(g) |
|  |  | caffeine 40 (mg/kg) | - | - | 99.87 ± 26.91 (U/L) | 111.80 ± 29.84 (U/L) | 437.30 ± 88.84* (U/L) | 512.10 ± 88.98 (U/L) | - | - | 7.48 ± 1.77 (g) |
|  |  | control | - | - | 139.75 ± 34.78 (U/L) | 146.75 ± 31.37 (U/L) | 713.75 ± 98.73 (U/L) | 616.13 ± 72.26 (U/L) | - | - | 8.50 ± 2.12 (g) |
| Xu, Zhang ^26^ | 2015 | caffeine | - | - | - | - | - | - | - | - | - |
|  |  | control | - | - | - | - | - | - | - | - | - |
| Kumbhar, Une ^27^ | 2016 | caffeine | - | - | 92 ± 3.7 (U/l) | 364 ± 16.4 (U/l) | - | - | - | - | - |
|  |  | control | - | - | 96 ± 3.5 (U/l) | 333 ± 19.4 (U/l) | - | - | - | - | - |
| Suzuki, Shindo ^8^ | 2017 | caffeine | - | - | - | - | - | - | - | - | - |
|  |  | control | - | - | - | - | - | - | - | - | - |
| Helal, Ayoub ^28^ | 2018 | caffeine 20 (mg/kg/ day) | - | - | 162 ± 11.7* (U/L) | 67 ± 2.1* (U/L) | - | - | 3.22 ± 0.04 (g/dL) | 0.4 ± 0.037* (mg/L) | - |
|  |  | caffeine 30 (mg/kg/ day) | - | - | 157.2 ± 5.7* (U/L) | 64 ± 4* (U/L) | - | - | 3.42 ± 0.09* (g/dL) | 0.4 ± 0.04* (mg/L) | - |
|  |  | control | - | - | 224 ± 20.5 (U/L) | 110.4 ± 3.2* (U/L) | - | - | 3.05 ± 0.07 (g/dL) | 0.66 ± 0.06 (mg/L) | - |
| Yang, Zhu ^29^ | 2019 | caffeine | - | - | 138 ± 14.5 | 63.6 ± 6.1* | - | - | - | - | - |
|  |  | control | - | - | 165.8 ± 17.3 | 77.3 ± 11.5 | - | - | - | - | - |

Insig = insignificant, sig = significant; * = p-value < 0.05 vs controls, ** = p-value < 0.01 vs controls, **^ = p-value < 0.005 vs controls, *** = p-value < 0.001 vs controls (higher or lower refers to the caffeine group vs control in that specific measure)

**Table E:** Insulin Resistance Outcome Measures Results

| Author | Year | Group Name | Insulin Resistance Outcome Measures | | | | | | | | | | | | | | | | | |
| --- | --- | --- | --- | --- | --- | --- | --- | --- | --- | --- | --- | --- | --- | --- | --- | --- | --- | --- | --- | --- |
|  |  |  | Fasting Plasma Glucose | Postprandial Plasma Glucose | Area Under Glucose Curve | Fasted Serum Insulin | Postprandial Serum Insulin | Area Under Insulin Curve | Fluid Intake (Ml/D) | Urine Volume (Ml/Dl) | Urine Glucose | Ogtt Intial | Ogtt 30m | Ogtt 60m | Ogtt 90 M | Ogtt 120m | ITT Intial | ITT 30m | Itt 60m | ITT 120m |
|  |  |  |  |  |  |  |  |  |  |  |  |  |  |  |  |  |  |  |  |  |
| Ohnishi A Fau - Branch, Branch Ra Fau - Jackson ^14^ | 1986 | caffeine | - | - | - | - | - | - | 32 ± 2 (mL/d) | 13 ± 1* (mL/dL) | - | - | - | - | - | - | - | - | - | - |
|  |  | control | - | - | - | - | - | - | 37 ± 2 (mL/d) | 20 ± 2 (mL/dL) | - | - | - | - | - | - | - | - | - | - |
| Sugiyama, Ohishi ^15^ | 1989 | caffeine | - | - | - | - | - | - | - | - | - | - | - | - | - | - | - | - | - | - |
|  |  | control | - | - | - | - | - | - | - | - | - | - | - | - | - | - | - | - | - | - |
| Choi, Lee ^16^ | 1993 | caffeine | - | - | - | - | - | - | - | - | - | - | - | - | - | - | - | - | - | - |
|  |  | control | - | - | - | - | - | - | - | - | - | - | - | - | - | - | - | - | - | - |
| Tofovic and Jackson ^17^ | 1999 | caffeine | - | - | - | - | - | - | - | - | - | - | - | - | - | - | - | - | - | - |
|  |  | control | - | - | - | - | - | - | - | - | - | - | - | - | - | - | - | - | - | - |
| Tanner and Tanner ^18^ | 2001 | caffeine 0.1 (mg/mL) | - | - | - | - | - | - | 25.1 ± 2.0 (mL/d/100 g body weight) | - | - | - | - | - | - | - | - | - | - | - |
|  |  | caffeine 0.2 (mg/mL) | - | - | - | - | - | - | 26.0 ± 3.8 (mL/d/100 g body weight) | - | - | - | - | - | - | - | - | - | - | - |
|  |  | control | - | - | - | - | - | - | 21.4 ± 4.9 (mL/d/100 g body weight) | - | - | - | - | - | - | - | - | - | - | - |
| Tofovic, Kusaka ^19^ | 2001 | caffeine | 145 ± 8.0 (mg/dL) | 283.3±19.6* (mg/dL) | 589.5 ± 20.5 (mg/dL × h) | 97.5±3.6 * (µIU/mL) | 110.6±3.4* (µIU/mL) | 198.0±5.9* (µIU/mL x h) | 79.5±5.0 * (mL/kg/d) | 72.9±3.3* (mL/kg/d) | 4.05±0.27* (mL/kg/d) | - | - | - | - | - | - | - | - | - |
|  |  | control | 138.7±13.4 (mg/dL) | 373±19.4 (mg/dL) | 592.5±42.7 (mg/dL × h) | 129.6±8.1 (µIU/mL) | 146.3±8.5 (µIU/mL) | 257.77±12.9 (µIU/mL x h) | 137.0±19.8 (mL/kg/d) | 109.6±15.1 (mL/kg/d) | 5.95±0.82 (mL/kg/d) | - | - | - | - | - | - | - | - | - |
| Tofovic, Kost ^20^ | 2002 | caffeine | 156±8*** (mg/dL) | - | - | 69.9±9.4*** (µU/mL) | - | - | 86±5*** (mL/kg/day) | 20.4±3.9*** (mL/min/g kidney) | 1.6±0.4**^ (g/day) | lower sig (p-value <0.05) | higher insig | lower insig | - | lower sig (p-value < 0.05) | - | - | - | - |
|  |  | control | 205±11 (mg/dL) | - | - | 88.2±6.0 (µU/mL) | - | - | 111±8 (mL/kg/day) | 33.5±3.7 (mL/min/g kidney) | 2.1±0.5 (g/day) | - | - | - | - | - | - | - | - | - |
| Park, Jang ^21^ | 2007 | caffeine | 6.6 ± 0.9 (mmol/L) | - | - | 357 ± 50 (pmol/L) | - | - | 67.8 ± 9.3 (mL/d) | - | - | - | - | - | - | - | - | - | - | - |
|  |  | control | 6.8 ± 0.8 (mmol/L) | - | - | 378 ± 53 (pmol/L) | - | - | 64.5 ± 8.5 (mL/d) | - | - | - | - | - | - | - | - | - | - | - |
| Tofovic, Salah ^22^ | 2007 | caffeine | - | - | lower sig (p-value <0.05) | 5.60 ± 0.53 (nano g/mL) | - | - | 122 ± 6* (mL/kg) | 114 ± 8* (mL/kg) | 11.4 ± 1.0* | lower sig (p-value <0.05) | lower insig | lower insig | - | lower sig (p-value < 0.05) | - | - | - | - |
|  |  | control | - | - | - | 6.76 ± 0.47 (nano g/mL) | - | - | 211 ± 20 (mL/kg) | 161 ± 12 (mL/kg) | 16.5 ± 3.3 | - | - | - | - | - | - | - | - | - |
| Kagami, Morita ^23^ | 2008 | caffeine 10 (mg/kg) | 159 ± 61 (mg/dL) | - | - | 0.15 ± 0.02 (nano g/mL) | - | - | - | - | - | lower insig | lower insig | lower sig (p-value <0.05) | - | - | - | - | - | - |
|  |  | caffeine 50 (mg/kg) | 105 ± 6** (mg/dL) | - | - | 0.20 ± 0.02 (nano g/mL) | - | - | - | - | - | lower insig | lower sig (p-value <0.01) | lower sig (p-value <0.001) | - | - | - | - | - | - |
|  |  | caffeine 100(mg/kg) | 94 ± 3** (mg/dL) | - | - | 0.17 ± 0.02 (nano g/mL) | - | - | - | - | - | lower sig (p-value <0.05) | lower sig (p-value <0.001) | lower sig (p-value <0.001) | - | - | - | - | - | - |
|  |  | control | 206 ± 36 (mg/dL) | - | - | 0.19 ± 0.028 (nano g/mL) | - | - | - | - | - | - | - | - | - | - | - | - | - | - |
| Conde, Nunes da Silva ^4^ | 2012 | Caffeine  HDFM | 1019 ± 40.7 (mg/L) | - | - | 1.84 ± 0.53*** (mg/L) | - | - | 6.01 ± 0.19 (mL/kg/d) | - | - | - | - | - | - | - | - | - | - | - |
|  |  | Control  HDFM | 1049 ± 26.2 (mg/L) | - | - | 5.48 ± 0.22 (mg/L) | - | - | 5.83 ± 0.25 (mL/kg/d) | - | - | - | - | - | - | - | - | - | - | - |
|  |  | Caffeine  HSDM | 1181 ± 48.6* (mg/L) | - | - | 4.74 ± 0.48 (mg/L) | - | - | 8.08 ± 0.68 (mL/kg/d) | - | - | - | - | - | - | - | - | - | - | - |
|  |  | Control  HSDM | 1458 ± 95.6 (mg/L) | - | - | 5.26 ± 0.28 (mg/L) | - | - | 8.31 ± 0.40 (mL/kg/d) | - | - | - | - | - | - | - | - | - | - | - |
| Panchal, Wong ^24^ | 2012 | caffeine | 4.1 ± 0.2* (mmol/L) | - | 562 ± 14* (mmol/L/min) | 0.65 ± 0.06 (pmol/L) | - | - | 19.5 ± 1.4 (mL/d) | - | - | 4.1 ± 0.2* (mmol/L) | lower insig | lower insig | - | lower sig (p-value <0.05) | lower insig | lower insig | lower insig | lower sig (p-value <0.05) |
|  |  | control | 5.0 ± 0.1 (mmol/L) | - | 771 ± 10 (mmol/L/min) | 0.55 ± 0.11 (pmol/L) | - | - | 18.8 ± 1.1 (mL/d) | - | - | 5.0 ± 0.1 (mmol/L) | - | - | - | - | - | - | - | - |
| Naidoo and Islam ^25^ | 2014 | caffeine 20 (mg/kg) | lower sig (p-value <0.05) | lower insig | - | 25.81 ± 5.57* (pmol/L) | - | - | lower insig | - | - | lower sig (p-value <0.05) | lower insig | lower insig | lower sig (p-value <0.05) | lower sig (p-value <0.05) | - | - | - | - |
|  |  | caffeine 40 (mg/kg) | lower sig (p-value <0.05) | lower insig | - | 21.53 ± 2.91* (pmol/L) | - | - | lower insig | - | - | lower sig (p-value <0.05) | lower insig | lower insig | lower insig | lower insig | - | - | - | - |
|  |  | control | - | - | - | 9.16 ± 1.64 (pmol/L) | - | - | - | - | - | - | - | - | - | - | - | - | - | - |
| Xu, Zhang ^26^ | 2015 | caffeine | - | - | - | - | - | - | - | - | - | - | - | - | - | - | - | - | - | - |
|  |  | control | - | - | - | - | - | - | - | - | - | - | - | - | - | - | - | - | - | - |
| Kumbhar, Une ^27^ | 2016 | caffeine | 485 ± 21 (mg/dL) | - | - | - | - | - | - | - | - | - | - | - | - | - | - | - | - | - |
|  |  | control | 474 ± 22 (mg/dL) | - | - | - | - | - | - | - | - | - | - | - | - | - | - | - | - | - |
| Suzuki, Shindo ^8^ | 2017 | caffeine | 107.8 ± 1.9*** (mg/dL) | - | - | 406.8 ± 82.3*** (pg/mL) | - | - | - | higher sig (p-value <0.05) | - | - | - | - | - | - | - | - | - | - |
|  |  | control | 259.5 ± 33.1 (mg/dL) | - | - | 1176.4 ± 157.4 (pg/mL) | - | - | - | - | - | - | - | - | - | - | - | - | - | - |
| Helal, Ayoub ^28^ | 2018 | caffeine 20 (mg/kg/ day) | - | - | - | - | - | - | - | - | - | - | - | - | - | - | - | - | - | - |
|  |  | caffeine 30 (mg/kg/ day) | - | - | - | - | - | - | - | - | - | - | - | - | - | - | - | - | - | - |
|  |  | control | - | - | - | - | - | - | - | - | - | - | - | - | - | - | - | - | - | - |
| Yang, Zhu ^29^ | 2019 | caffeine | - | - | - | - | - | - | - | - | - | - | - | - | - | - | - | - | - | - |
|  |  | control | - | - | - | - | - | - | - | - | - | - | - | - | - | - | - | - | - | - |

Insig = insignificant, sig = significant; * = p-value < 0.05 vs controls, ** = p-value < 0.01 vs controls, **^ = p-value < 0.005 vs controls, *** = p-value < 0.001 vs controls (higher or lower refers to the caffeine group vs control in that specific measure)

**Table F:** Hypertension Outcome Measures Results

| Author | Year | Group Name | Hypertension Outcome Measures | | |
| --- | --- | --- | --- | --- | --- |
|  |  |  | **Systolic Blood Pressure** | **Diastolic Blood Pressure** | **Mean Arterial Blood Pressure** |
|  |  |  |  |  |  |
| Ohnishi A Fau - Branch, Branch Ra Fau - Jackson ^14^ | 1986 | caffeine | lower insig | - | - |
|  |  | control | - | - | - |
| Sugiyama, Ohishi ^15^ | 1989 | caffeine | - | - | - |
|  |  | control | - | - | - |
| Choi, Lee ^16^ | 1993 | caffeine | lower sig (p-value <0.01) | - | - |
|  |  | control | - | - | - |
| Tofovic and Jackson ^17^ | 1999 | caffeine | 181.4 ± 10.9 (mmHg) | 151.4 ± 9.1 (mmHg) | 161.8 ± 8.6 (mmHg) |
|  |  | control | 192.9 ± 5.5 (mmHg) | 166.1 ± 4 (mmHg) | 179.9 ± 4 (mmHg) |
| Tanner and Tanner ^18^ | 2001 | caffeine 0.1 (mg/mL) | - | - | 124 ± 7* (mmHg) |
|  |  | caffeine 0.2 (mg/mL) | - | - | 139 ± 16*** (mmHg) |
|  |  | control | - | - | 117 ± 10 (mmHg) |
| Tofovic, Kusaka ^19^ | 2001 | caffeine | 187.8 ± 5.2 (mmHg) | 143.5 ± 2.3 (mmHg) | 166.0 ± 3.4 (mmHg) |
|  |  | control | 181.5 ± 5.0 (mmHg) | 140.7 ± 3.6 (mmHg) | 160.7 ± 4.1 (mmHg) |
| Tofovic, Kost ^20^ | 2002 | caffeine | higher sig (p-value <0.05) | higher sig (p-value <0.05) | higher sig (p-value <0.05) |
|  |  | control | - | - | - |
| Park, Jang ^21^ | 2007 | caffeine | - | - | - |
|  |  | control | - | - | - |
| Tofovic, Salah ^22^ | 2007 | caffeine | - | - | 159.8 ± 4.0 (mmHg) |
|  |  | control | - | - | 149.5 ± 2.8 (mmHg) |
| Kagami, Morita ^23^ | 2008 | caffeine 10 (mg/kg) | - | - | - |
|  |  | caffeine 50 (mg/kg) | - | - | - |
|  |  | caffeine 100(mg/kg) | - | - | - |
|  |  | control | - | - | - |
| Conde, Nunes da Silva ^4^ | 2012 | Caffeine  HFDM | - | - | 86.92 ± 3.96** (mmHg) |
|  |  | Control  HFDM | - | - | 108.04 ± 5.30 mmHg |
|  |  | Caffeine  HSDM | - | - | 92.13 ± 1.82* (mmHg) |
|  |  | Control  HSDM | - | - | 104.69 ± 2.72 (mmHg) |
| Panchal, Wong ^24^ | 2012 | caffeine | 118 ± 1* (mmHg) | - | - |
|  |  | control | 145 ± 1 (mmHg) | - | - |
| Naidoo and Islam ^25^ | 2014 | caffeine 20 (mg/kg) | - | - | - |
|  |  | caffeine 40 (mg/kg) | - | - | - |
|  |  | control | - | - | - |
| Xu, Zhang ^26^ | 2015 | caffeine | - | - | - |
|  |  | control | - | - | - |
| Kumbhar, Une ^27^ | 2016 | caffeine | - | - | - |
|  |  | control | - | - | - |
| Suzuki, Shindo ^8^ | 2017 | caffeine | 141.9 ± 2.7** (mmHg) | 94.2 ± 5.3* (mmHg) | - |
|  |  | control | 154.2 ± 2.8 (mmHg) | 110.1 ± 3.1 (mmHg) | - |
| Helal, Ayoub ^28^ | 2018 | caffeine 20 (mg/kg/ day) | - | - | - |
|  |  | caffeine 30 (mg/kg/ day) | - | - | - |
|  |  | control | - | - | - |
| Yang, Zhu ^29^ | 2019 | caffeine | - | - | - |
|  |  | control | - | - | - |

Insig = insignificant, sig = significant; * = p-value < 0.05 vs controls, ** = p-value < 0.01 vs controls, **^ = p-value < 0.005 vs controls, *** = p-value < 0.001 vs controls (higher or lower refers to the caffeine group vs control in that specific measure)

**Table G:** Prisma checklist

| **Section and Topic** | **Item #** | **Checklist item** | **Location where item is reported** |
| --- | --- | --- | --- |
| **TITLE** | | | Page 1 |
| Title | 1 | Identify the report as a systematic review. | Page 1 |
| **ABSTRACT** | | |  |
| Abstract | 2 | See the PRISMA 2020 for Abstracts checklist. | Page 1 |
| **INTRODUCTION** | | |  |
| Rationale | 3 | Describe the rationale for the review in the context of existing knowledge. | Page 2 |
| Objectives | 4 | Provide an explicit statement of the objective(s) or question(s) the review addresses. | Page 2 |
| **METHODS** | | |  |
| Eligibility criteria | 5 | Specify the inclusion and exclusion criteria for the review and how studies were grouped for the syntheses. | Page 2 |
| Information sources | 6 | Specify all databases, registers, websites, organisations, reference lists and other sources searched or consulted to identify studies. Specify the date when each source was last searched or consulted. | Page 2 |
| Search strategy | 7 | Present the full search strategies for all databases, registers and websites, including any filters and limits used. | Page 2 |
| Selection process | 8 | Specify the methods used to decide whether a study met the inclusion criteria of the review, including how many reviewers screened each record and each report retrieved, whether they worked independently, and if applicable, details of automation tools used in the process. | Page 3 |
| Data collection process | 9 | Specify the methods used to collect data from reports, including how many reviewers collected data from each report, whether they worked independently, any processes for obtaining or confirming data from study investigators, and if applicable, details of automation tools used in the process. | Page 3 |
| Data items | 10a | List and define all outcomes for which data were sought. Specify whether all results that were compatible with each outcome domain in each study were sought (e.g. for all measures, time points, analyses), and if not, the methods used to decide which results to collect. | Page 3 |
|  | 10b | List and define all other variables for which data were sought (e.g. participant and intervention characteristics, funding sources). Describe any assumptions made about any missing or unclear information. | Page 3 |
| Study risk of bias assessment | 11 | Specify the methods used to assess risk of bias in the included studies, including details of the tool(s) used, how many reviewers assessed each study and whether they worked independently, and if applicable, details of automation tools used in the process. | Page 3 |
| Effect measures | 12 | Specify for each outcome the effect measure(s) (e.g. risk ratio, mean difference) used in the synthesis or presentation of results. | Page 3 |
| Synthesis methods | 13a | Describe the processes used to decide which studies were eligible for each synthesis (e.g. tabulating the study intervention characteristics and comparing against the planned groups for each synthesis (item #5)). | Page 4 |
|  | 13b | Describe any methods required to prepare the data for presentation or synthesis, such as handling of missing summary statistics, or data conversions. | Page 4 |
|  | 13c | Describe any methods used to tabulate or visually display results of individual studies and syntheses. | Page 4 |
|  | 13d | Describe any methods used to synthesize results and provide a rationale for the choice(s). If meta-analysis was performed, describe the model(s), method(s) to identify the presence and extent of statistical heterogeneity, and software package(s) used. | Page 4 |
|  | 13e | Describe any methods used to explore possible causes of heterogeneity among study results (e.g. subgroup analysis, meta-regression). | Page 4 |
|  | 13f | Describe any sensitivity analyses conducted to assess robustness of the synthesized results. | - |
| Reporting bias assessment | 14 | Describe any methods used to assess risk of bias due to missing results in a synthesis (arising from reporting biases). | Page 3 |
| Certainty assessment | 15 | Describe any methods used to assess certainty (or confidence) in the body of evidence for an outcome. | Page 4 |
| **RESULTS** | | |  |
| Study selection | 16a | Describe the results of the search and selection process, from the number of records identified in the search to the number of studies included in the review, ideally using a flow diagram. | Page 5 |
|  | 16b | Cite studies that might appear to meet the inclusion criteria, but which were excluded, and explain why they were excluded. | Page 5 |
| Study characteristics | 17 | Cite each included study and present its characteristics. | Page 5 |
| Risk of bias in studies | 18 | Present assessments of risk of bias for each included study. | Page 4 |
| Results of individual studies | 19 | For all outcomes, present, for each study: (a) summary statistics for each group (where appropriate) and (b) an effect estimate and its precision (e.g. confidence/credible interval), ideally using structured tables or plots. | Page 5 |
| Results of syntheses | 20a | For each synthesis, briefly summarise the characteristics and risk of bias among contributing studies. | Page 5 |
|  | 20b | Present results of all statistical syntheses conducted. If meta-analysis was done, present for each the summary estimate and its precision (e.g. confidence/credible interval) and measures of statistical heterogeneity. If comparing groups, describe the direction of the effect. | - |
|  | 20c | Present results of all investigations of possible causes of heterogeneity among study results. | Page 4 |
|  | 20d | Present results of all sensitivity analyses conducted to assess the robustness of the synthesized results. | - |
| Reporting biases | 21 | Present assessments of risk of bias due to missing results (arising from reporting biases) for each synthesis assessed. | Page 4 |
| Certainty of evidence | 22 | Present assessments of certainty (or confidence) in the body of evidence for each outcome assessed. | Page 4 |
| **DISCUSSION** | | |  |
| Discussion | 23a | Provide a general interpretation of the results in the context of other evidence. | Page 10 |
|  | 23b | Discuss any limitations of the evidence included in the review. | Page 12 |
|  | 23c | Discuss any limitations of the review processes used. | Page 12 |
|  | 23d | Discuss implications of the results for practice, policy, and future research. | Page 12 |
| **OTHER INFORMATION** | | |  |
| Registration and protocol | 24a | Provide registration information for the review, including register name and registration number, or state that the review was not registered. | Page 2 |
|  | 24b | Indicate where the review protocol can be accessed, or state that a protocol was not prepared. | Page 2 |
|  | 24c | Describe and explain any amendments to information provided at registration or in the protocol. | Page 2 |
| Support | 25 | Describe sources of financial or non-financial support for the review, and the role of the funders or sponsors in the review. | Page 13 |
| Competing interests | 26 | Declare any competing interests of review authors. | Page 13 |
| Availability of data, code and other materials | 27 | Report which of the following are publicly available and where they can be found: template data collection forms; data extracted from included studies; data used for all analyses; analytic code; any other materials used in the review. | Page 13 |

*From:*  Page MJ, McKenzie JE, Bossuyt PM, Boutron I, Hoffmann TC, Mulrow CD, et al. The PRISMA 2020 statement: an updated guideline for reporting systematic reviews. BMJ 2021;372:n71. doi: 10.1136/bmj.n71

For more information, visit: <http://www.prisma-statement.org/>
